# An analysis framework for the integration of broadband NIRS and EEG to assess neurovascular and neurometabolic coupling

**DOI:** 10.1038/s41598-021-83420-9

**Published:** 2021-02-17

**Authors:** P. Pinti, M. F. Siddiqui, A. D. Levy, E. J. H. Jones, Ilias Tachtsidis

**Affiliations:** 1 Department of Medical Physics and Biomedical Engineering, University College London, London, UK; 2 Centre for Brain and Cognitive Development, Birkbeck College, University of London, London, UK; 3 Wellcome Centre for Human Neuroimaging, Institute of Neurology, University College London, London, UK; 4 Headache and Facial Pain, Brain Repair and Rehabilitation, Institute of Neurology, University College London, London, UK

**Keywords:** Cognitive neuroscience, Near-infrared spectroscopy

## Abstract

With the rapid growth of optical-based neuroimaging to explore human brain functioning, our research group has been developing broadband Near Infrared Spectroscopy (bNIRS) instruments, a technological extension to functional Near Infrared Spectroscopy (fNIRS). bNIRS has the unique capacity of monitoring brain haemodynamics/oxygenation (measuring oxygenated and deoxygenated haemoglobin), *and* metabolism (measuring the changes in the redox state of cytochrome-c-oxidase). When combined with electroencephalography (EEG), bNIRS provides a unique neuromonitoring platform to explore neurovascular coupling mechanisms. In this paper, we present a novel pipeline for the integrated analysis of bNIRS and EEG signals, and demonstrate its use on multi-channel bNIRS data recorded with concurrent EEG on healthy adults during a visual stimulation task. We introduce the use of the Finite Impulse Response functions within the General Linear Model for bNIRS and show its feasibility to statistically localize the haemodynamic and metabolic activity in the occipital cortex. Moreover, our results suggest that the fusion of haemodynamic and metabolic measures unveils additional information on brain functioning over haemodynamic imaging alone. The cross-correlation-based analysis of interrelationships between electrical (EEG) and haemodynamic/metabolic (bNIRS) activity revealed that the bNIRS metabolic signal offers a unique marker of brain activity, being more closely coupled to the neuronal EEG response.

## Introduction

Over the past few decades, neuroimaging technologies have allowed us to advance our knowledge of the mechanisms underlying human brain function, both in health and disease. Functional neuroimaging methodologies are widely used in the fields of cognitive neuroscience and neuropsychology in order to investigate the relationship between a specific task or mental function and the corresponding patterns and/or locations of brain activity. Different functional neuroimaging techniques are based on different principles, and can be broadly categorized as: electrophysiological (e.g., electroencephalography (EEG) and magnetoencephalography (MEG)), haemodynamic (e.g., functional magnetic resonance spectroscopy (fMRI) and functional near-infrared spectroscopy (fNIRS)), and metabolic (e.g. fluorodeoxyglucose positron emission tomography (FDG-PET) and broadband near infrared spectroscopy (bNIRS)). Each of these capture different and complimentary aspects of brain function, distinguished by the complex sequence of neurophysiology that underlies the generation of their signals. Furthermore, each modality has strengths and limitations that largely depend on the spatiotemporal characteristics of the signal. Electrophysiological recordings provide *direct* measures of neuronal activity with high temporal resolution but poor spatial resolution, while haemodynamic and metabolic imaging methods are able to *indirectly* assess brain activity by capturing the physiological changes linked to neurovascular and neurometabolic coupling, with better spatial specificity but lower temporal resolution. It has therefore become increasing popular to fuse data from multiple imaging modalities to yield a more detailed understanding of brain dynamics.

Among these, the use of optical methods, and particularly fNIRS is growing exponentially^1^. This growth can be attributed to its suitability for use with a wide range of populations and experiments in more ecological settings not afforded by other imaging modalities (i.e., portability, low sensitivity to movements, safety; see Pinti et al.^2^ for a review^2^) and for multimodal imaging (i.e., ease of integration and lack of interference with other neuroimaging methods). fNIRS measures the changes in brain tissue concentration of oxygenated (HbO_2_) and deoxygenated (HHb) haemoglobin. These in turn reflect the changes in cerebral blood flow (CBF) that follows neuronal activation, a physiological phenomenon referred to as neurovascular coupling^3^. When a brain area becomes active and neurons increase their rate of firing in response to a certain task, an increase in the metabolic demand for oxygen and glucose is observed in that area. This metabolic demand causes an oversupply of CBF in compensation. Therefore, we observe an increase in HbO_2_ and tissue metabolism and a decrease in HHb following neuronal firing. fNIRS measurements are obtained by shining near infrared (NIR) light (650–950 nm) into the head and collecting the backscattered light to quantify the changes in light absorbance; the changes in HbO_2_ and HHb concentrations are then derived using the modified Beer-lambert law (MBLL) algorithm. The current commercially available instruments are equipped with several light sources and detectors, i.e. *multi-channel*, allowing simultaneous measurements at different locations on the head.

Light sources used in traditional NIRS systems typically emit light at 2 or 3 wavelengths in the NIR range, which allow users to resolve changes in HbO_2_ and HHb concentrations only. However, for several years our team has been developing a technological and methodological extension to fNIRS called broadband NIRS (bNIRS). bNIRS is an optical-based neuroimaging technology that utilizes hundreds of NIR wavelengths with the capacity to measure an additional brain tissue chromophore, cytochrome-c-oxidase or CCO. CCO is the terminal enzyme of the electron transport chain in the mitochondria and is responsible for catalysing 95% of oxygen to produce energy in the form of ATP; CCO thus provides an indicator of cerebral cellular oxygen metabolism. bNIRS can quantify the redox state of cytochrome-c-oxidase (oxCCO), that contributes to the NIR spectrum of the brain tissue together with haemoglobin. Absorption in the NIR range, with a peak at ~ 830 nm, is dominated by one of the CCO copper sites in its oxidized form (oxCCO). This signal can be measured using bNIRS. For a complete overview on bNIRS-based measurements of oxCCO, see Bale and colleagues (2016) for review. Measuring oxCCO presents several challenges. For instance, oxygenation measurements are easier to perform as HbO_2_ and HHb are present in higher concentration than CCO. HbO_2_ and HHb signals can hence mask oxCCO signals and proper algorithms, wavelengths and extinction spectra are necessary to avoid cross talk and ensure that changes in all three chromophores are properly resolved^4^. However, it has been demonstrated that oxCCO can provide more specific brain signals than haemoglobin^5^. This may be due to the fact that CCO is more concentrated in the intracerebral compartment^6^ and is less contaminated by the extracerebral layers, while haemoglobin signals can be strongly confounded by systemic contamination from the extracerebral layers^7^. A previous study by de Roever and colleagues has demonstrated this further by investigating both haemodynamic and metabolic activity during a functional task at different depths and confirming that higher oxCCO responses can be found in deeper channels rather than in the superficial ones, while haemodynamic responses were observed in all the channels^8^.

The development of bNIRS instruments using the full spectrum of NIR light and novel algorithms (i.e., ‘UCLn’^9^) have aided in overcoming some of the aforementioned technical issues and can robustly estimate both haemoglobin and oxCCO changes. Therefore, bNIRS presents a truly unique method to monitor both the changes in brain oxygenation and haemodynamics (∆HbO_2_ and ∆HHb) *and* in metabolism (∆oxCCO) induced by neuronal activation *simultaneously* and *non-invasively* (Note: For simplicity, we will use ‘brain haemodynamics’ to refer to ‘brain oxygenation and haemodynamics measurements’ throughout the manuscript).

The possibility of assessing how the energy is supplied and used by the brain under different conditions paves the way for novel fundamental investigations of the mechanisms underlying neurovascular coupling and has the potential to advance our understanding of brain physiology and cognitive functioning both in health and in disease. Previous studies have suggested that, in order to properly understand the functioning of the brain and its alteration, it is important to consider not only the differences in metabolism across regions but to also account for the underlying brain activity. It has also been demonstrated that high neuronal activity is typically associated to high metabolic supply in a healthy brain, and that brain regions may show different relationships between activity demand and metabolic supply (e.g., metabolism may fall behind neuronal activity in a certain region depending on which metabolic substrate is used), which in turn may be altered in case of neuropsychiatric disorders or disease^10^. For instance, Shokri-Kojori and colleagues (2019) have recently demonstrated how alcohol intoxication can alter the relationship between neuronal activity and glucose metabolism as measured by fluorodeoxyglucose-positron emission tomography (FDG-PET) and fMRI. However, gold standard neuroimaging technologies such as PET and fMRI do not allow the monitoring of both metabolic and neuronal activity of the brain simultaneously and separate testing sessions are required, which in turn can be problematic for clinical populations. Here, we propose that bNIRS can be an ideal candidate to gather data on both the brain metabolic supply and oxygenation/haemodynamic changes and to evaluate their relationship simultaneously and non-invasively.

To date, there are no commercially available bNIRS instruments and existing systems are developed entirely in laboratories^11,12^. Therefore, the use of bNIRS is not as popular as fNIRS yet. Few functional activation studies with bNIRS have been performed on adults (see Bale et al.^4^ for review)^6,8,13–16^ and only two in infants^17,18^. In particular, conventional stimulation protocols have mainly been employed thus far, such as visual^14^ and auditory^15^ stimulation or anagram solving tasks^6,13^. Results from these studies were derived using basic analytical approaches, such as block-averaging combined with t-tests to infer functional activity at a group level^6,8,13–17^. However, none of these basic statistical approaches utilizes and take into consideration the haemodynamic and metabolic response and, importantly, their interrelationship. Moreover, the coupling of neural and vascular responses (also known as neurovascular coupling) is not very well understood, particularly in pathology, primarily due to the lack of availability of feasible techniques. While the measurement of haemodynamic and metabolic activity through the use of bNIRS systems provide an understanding on one end of neurovascular coupling, investigation still needs to be done to understand how neural electrical activity relates to metabolic and haemodynamic activity in order to obtain a more holistic understanding of basic biological mechanisms in the brain. EEG offers a measurement of neuronal functioning by measuring the brain electrical activity directly. Therefore, the combination of bNIRS and EEG has the potential to provide enhanced and holistic information on neurovascular coupling and a window into brain oxygenation, haemodynamics, metabolism and neuronal function. Previous studies have combined fMRI and EEG for the purposes of this exploration^19–22^. Studies have also combined fNIRS with EEG to investigate neurovascular coupling in both clinical and non-clinical applications (see Chiarelli et al.^23^ for review) and have demonstrated the strength of fNIRS-EEG as multimodal tool to assess both the brain electrical and haemodynamic activity. However, the haemodynamic and electrical signals measured by fNIRS and EEG do not have exact spatiotemporal correspondence^23^ and have very different time resolution (order of seconds for fNIRS, order of milliseconds for EEG). Therefore, the integration of haemodynamic data with EEG presents several challenges and new methods need to be explored. In addition, previous works did not include bNIRS derived metabolic measures and thus did not evaluate the relationship between brain neuronal and metabolic activity.

Generally, statistical modelling procedures are commonly employed in functional neuroimaging studies^24^ to characterize and localize the neural responses to cognitive tasks and to infer functional activity. They usually make use of General Linear Models (GLMs)^25^ to deal with the complex characteristics of functional neuroimaging data. The canonical GLM is a well-established regression approach extensively used for haemodynamic-based neuroimaging data analysis, such as for fMRI^25^ and fNIRS^26^. It is able to capture cerebral activity by fitting the experimental data with a model of functional activity built through the convolution of a boxcar function, describing the experimental protocol, with a Haemodynamic Response Function (HRF)^25^. The canonical HRF reflects the changes in CBF in response to neuronal activation and is usually modelled as a linear combination of two Gamma functions. The advancements in neuroimaging statistical methodologies have made possible to have freeware software platforms implementing processing and canonical GLM-based statistical analyses of fNIRS haemodynamic data (e.g., Ye et al.^27^^27^ or Sutoko et al.^28^^28^) but these do not take into consideration metabolic signals and deal only with HbO_2_ and HHb. In addition, there is no analogous of the HRF for oxCCO at the moment. Therefore, the existing statistical framework needs to be expanded for bNIRS to make inferences on both haemodynamic *and* metabolic neuroimaging data. For instance, de Roever and colleagues (2017) experimented with the use of deconvolution GLM using Gaussian basis function with no a-priori assumption on the shape of the haemodynamic and metabolic responses for short-separation channel regression (i.e., remove the extra-cerebral interference from long separation channels). Results showed that this method was suitable to recover both the haemodynamic and metabolic responses while at the same time removing scalp contamination as measured by short-separation channels^29^.

With the advent of the bNIRS technology, which provides the capacity for imaging both brain haemodynamics *and* metabolism (HbO_2_, HHb, oxCCO), there is an urgent need for new methods and tools that will enable extension of the more advanced statistical approaches typically used for haemodynamic neuroimaging data to the analysis of bNIRS-derived metabolic signals. In this paper, we demonstrate the use of bNIRS to monitor the haemodynamic and metabolic activity of the visual cortex of healthy adults and we establish a new analysis framework for the localization of brain activity and the fusion of haemodynamics with metabolic and neuronal information. In particular, we present a novel analysis pipeline that allows us to (i) pre-process and denoise the bNIRS signals, (ii) construct a statistical framework that can be used to test hypothesis about functional haemodynamic *and* metabolic imaging data (iii) combine the information derived from the analysis of haemodynamic and metabolic data to assess the relationship between the brain haemodynamics and metabolism during functional activity, and (iv) construct a data analysis pipeline that allows the integration of bNIRS signals with EEG in order to obtain a more holistic understanding of neurovascular coupling mechanisms. Our analysis pipeline: (1) includes steps to minimize physiological noise and motion artifacts; (2) is based on the GLM framework employing FIR basis functions to overcome the issue of making assumptions on the models of haemodynamic and metabolic responses; (3) expands on the use of two indices reflecting the correspondence between cerebral metabolism and brain haemodynamics (relative power and relative cost) recently developed by Shokri-Kojori and colleagues (2019) using FDG-PET and fMRI^10^ to measures extracted from bNIRS data; (4) assesses the similarity and time dynamic relationship between the bNIRS and EEG gamma band activity. We demonstrate the use of the proposed pipeline on bNIRS data recorded on healthy adults during a visual stimulation task.

## Results

### bNIRS pre-processing

**Figure 1:**
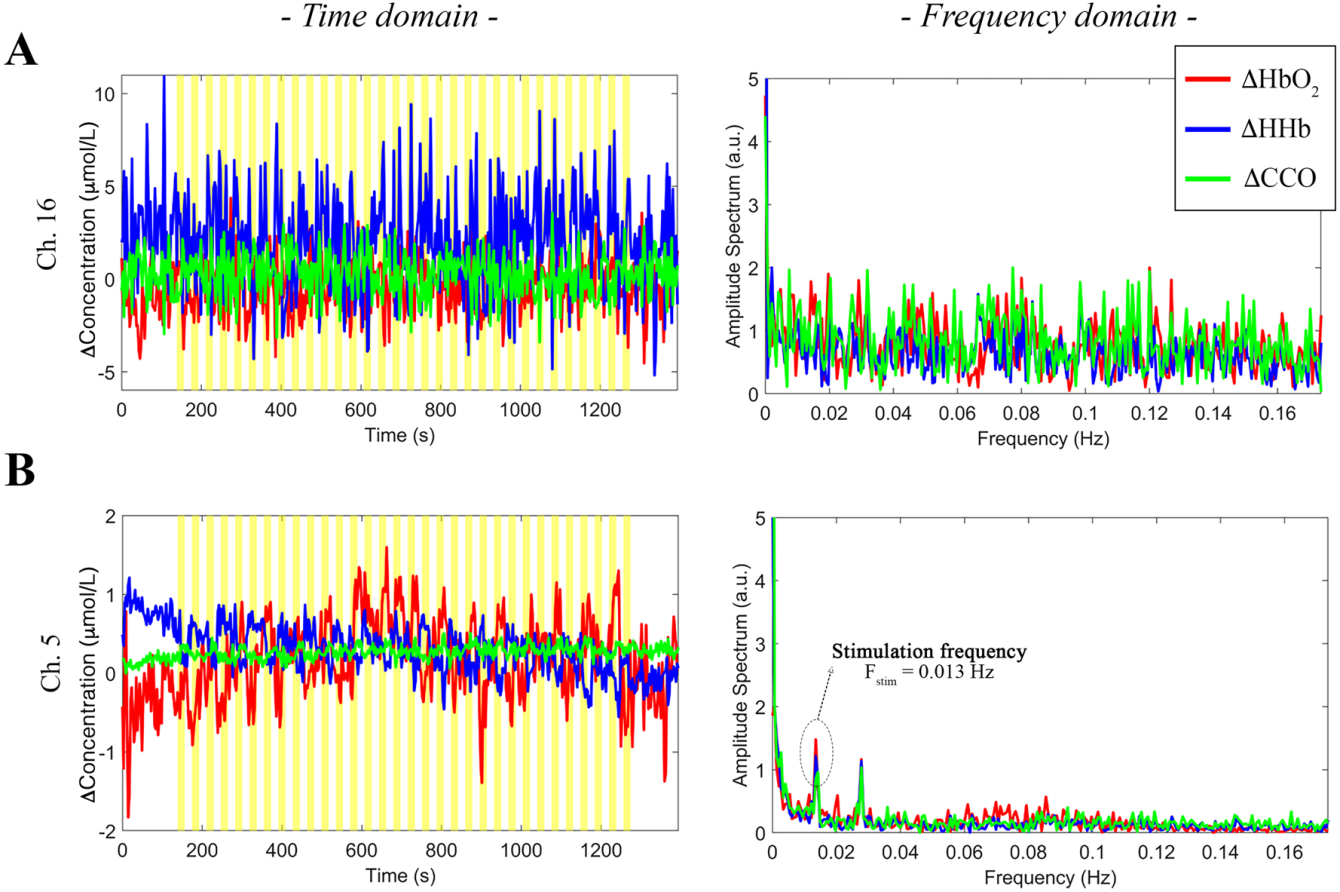
Examples of poor quality (**A**) and good quality (**B**) ΔHbO_2_ (red), ΔHHb (blue), and ΔoxCCO (green) signals of two channels from one participant. Data are shown both in the time domain (left) and in the frequency domain (right). Yellow areas mark the visual task blocks.

Following the conversion of the raw intensity spectra into raw concentration changes (Phase 1, Fig. 10), we visually inspected the raw ΔHbO_2_, ΔHHb, and ΔoxCCO signals to identify the channels with a low signal to noise ratio. An example of a noisy channel from one participant is shown in Fig. 1A: where the time-series data exhibit higher than normal amplitude changes (≥ 5 mol/L) particularly for HHb and oscillations appear random and not task-related; their frequency spectra indeed resemble the spectrum of white noise, suggesting that the optical coupling between the optode and the head was not optimal in this case^30^.

In Fig. 1B, the raw ΔHbO_2_, ΔHHb, and ΔoxCCO from a good quality channel from the same participant is shown, where the signals’ changes are within a more physiological range (~ 1–1.5 mol/L), and the corresponding FFTs have the typical 1/f decreasing trend and the task-related frequency can be clearly identified around 0.014 Hz (F_stim_ = 1/72 s; i.e. every 72 s a new hemifield stimulus was presented) for all three chromophores.

Following this procedure, we excluded channel 13, 15 and 16 for one participant as no heart rate component could be detected and the signals’ characteristics resembled white noise features. All channels were included for the rest of our cohort, suggesting that the 3D printed optode holder was effective in maximizing signals quality and the optimal coupling while minimizing the holder displacements.

**Figure 2:**
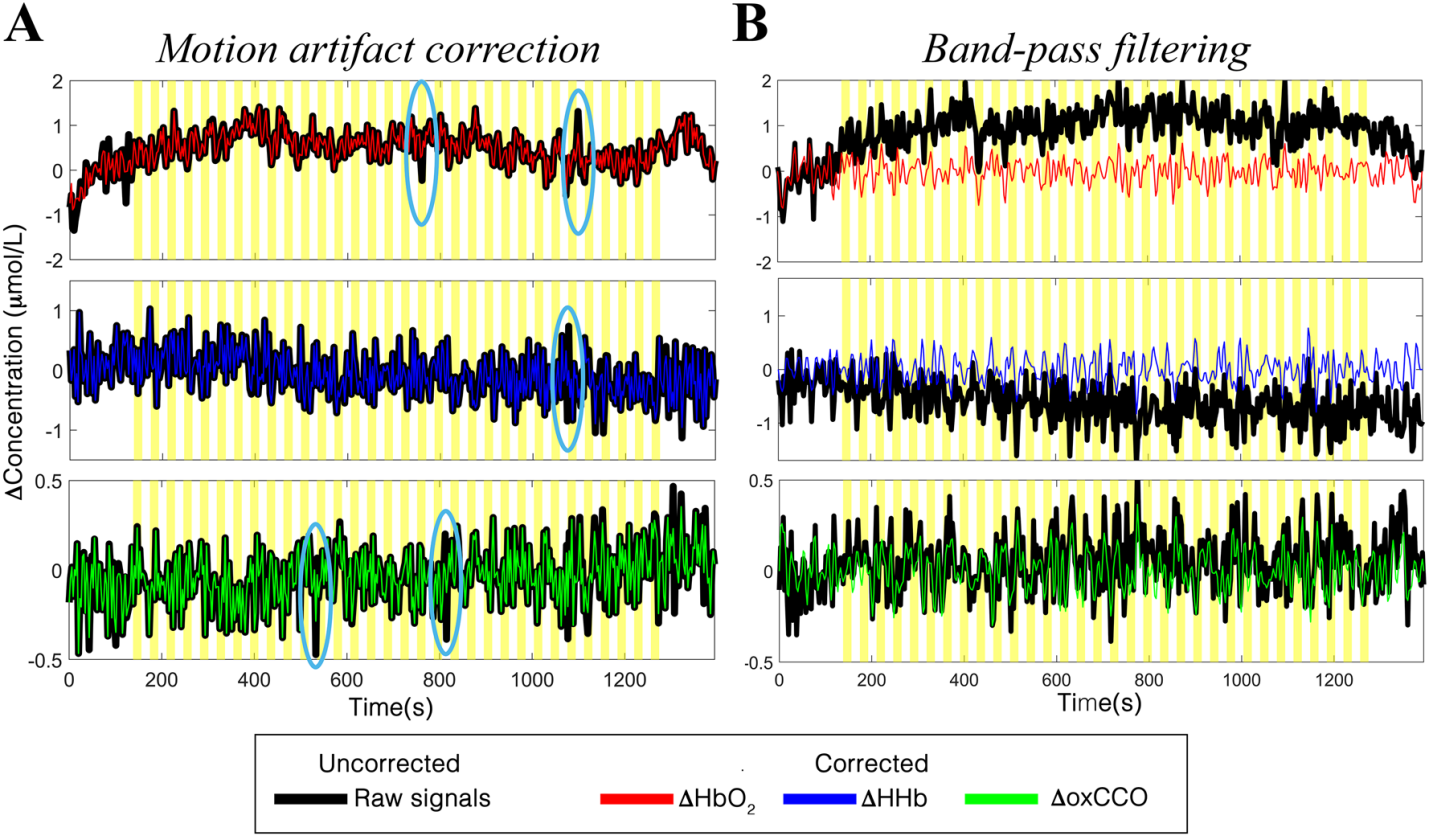
Examples of the result of the application of the preprocessing steps to the ΔHbO_2_ (top), ΔHHb (middle), and ΔoxCCO (bottom) signals of one participant. Panel (**A**) shows the application of motion artifact correction method, where examples of artifacts are circled in light blue. Panel (**B**) shows the results of the FIR band-pass filtering. Raw uncorrected ΔHbO_2_/ΔHHb,/ΔoxCCO are shown in black,; processed ΔHbO_2_, ΔHHb, and ΔoxCCO are shown in red, blue and green respectively. Yellow areas mark the visual task blocks.

The preprocessing steps successfully allowed minimization of the unwanted noisy components in our data. In terms of motion artifacts, an example of the application of the wavelet-based method to our haemodynamic and metabolic data of one participant is shown in Fig. 2A.

It can be observed that the wavelet filtering was able to properly correct the fast spikes due to rapid head movements and temporary displacement of the optodes while preserving the haemodynamic/metabolic modulations of our signals. In addition, the band-pass filter was effective in removing both the slow trends in the data and smoothing the higher frequencies (Fig. 2B), with the final chromophore signals centered around the zero-level and with an amplitude comprised between -1 and 1 µmol/L that are typical concentration changes during functional brain activity^31^.

### bNIRS statistical analysis

**Figure 3:**
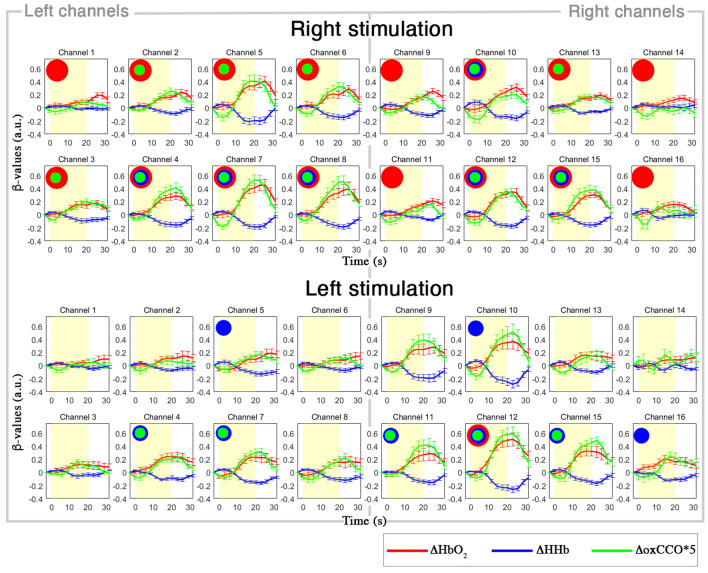
Grand-averaged responses (mean ± std. error) for the Right (top) and Left (bottom) visual hemifield condition, for each channel. Single-subject responses are estimated through the FIR-GLM and averaged across the group. The yellow area indicates the task block. ΔHbO_2_ is shown in red, ΔHHb in blue, ΔoxCCO (magnified by a factor of 5 for better visualization) in green. Red circles represent statistically significant ΔHbO_2_ changes, blue circles statistically significant ΔHHb changes, and green circles statistically significant ΔoxCCO changes (p < 0.005, FDR corrected for multiple comparisons).

In Fig. 3, we present the results of the application of the FIR-based GLM to ΔHbO_2_, ΔHHb, and ΔoxCCO. In particular, the group-averaged estimated responses ΔHbO_2_, ΔHHb, and ΔoxCCO are shown for all the channels for the Right and Left experimental conditions.

These results demonstrate that the FIR basis functions are able to capture the haemodynamic and metabolic responses to our functional task, recovering an average response with the typical trend of fNIRS data recorded with block-designed experiments (i.e., increase in ΔHbO_2_ and decrease in ΔHHb)^2^ and with larger changes in the contralateral channels to the visual stimulus. To localize functional brain activity at the group level, we carried out a channel-wise one sample *t*-tests against 0 on the median β2-values values extracted from the ΔHbO_2_, ΔHHb, and ΔoxCCO estimated responses. Group-level results are included in Fig. 3, where red circles represent statistically significant ΔHbO_2_ changes, blue circles statistically significant ΔHHb changes, and green circles statistically significant ΔoxCCO changes (p < 0.005, FDR corrected for multiple comparisons). Group-level *t*-values for each chromophore and channel are reported in Supplementary Table 2.

### Integration of bNIRS haemodynamic and metabolic data

**Figure 4:**
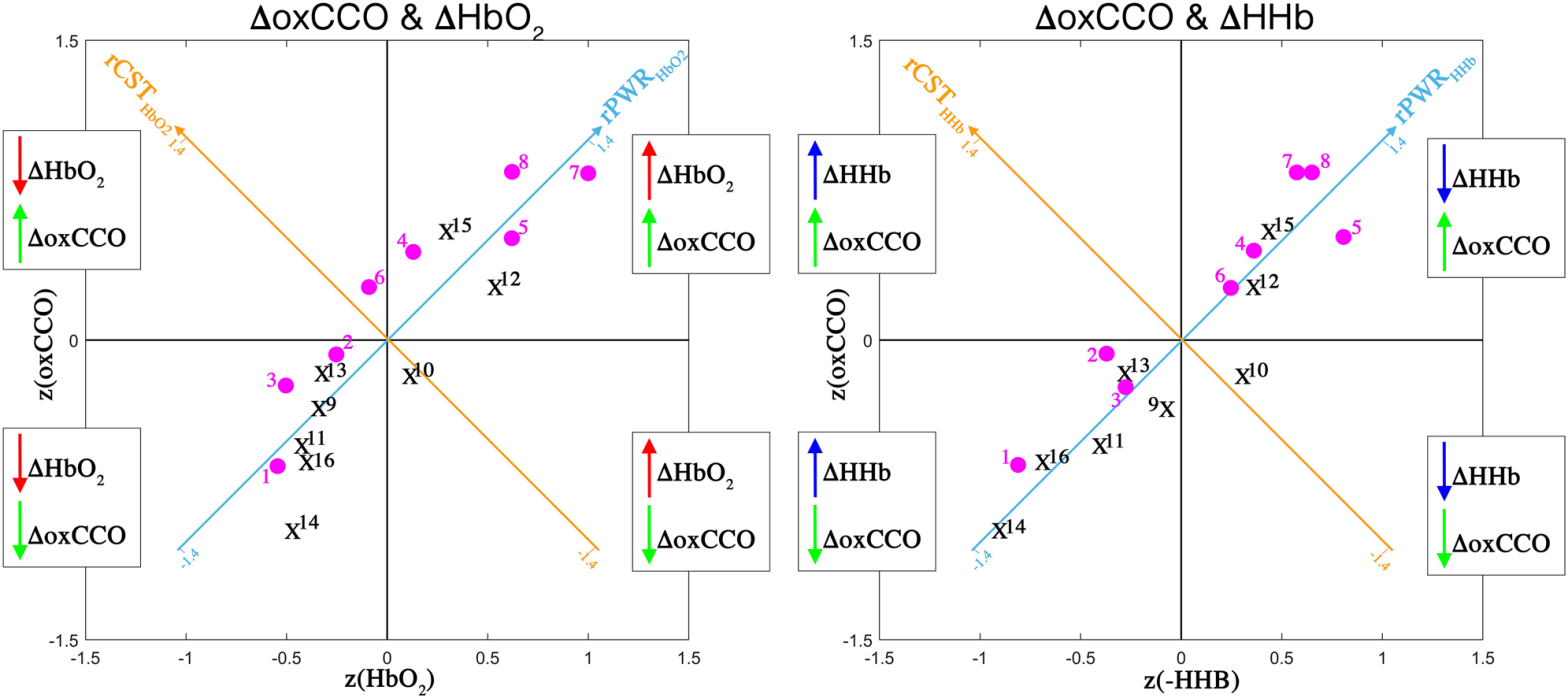
Visual representation of rPWR and rCST for the Right condition. These parameters were computed from the median β-values which were z-scored across channels and then averaged across all participants, for HbO_2_ and oxCCO (left) and HHb and oxCCO (right) by rotating of 45° along the haemodynamics and metabolism axis, yielding to a rPWR axis (light blue) and rCST axis (orange). bNIRS channels located in the left hemisphere (# 1–8) are indicated by magenta circles and black crosses indicate channels located in the right hemisphere (# 9–16). The map is divided into four quadrants based on the direction and relative magnitude of the chromophores’ changes (greater increase or decrease) across channels.

To evaluate the relationship between brain haemodynamics and metabolism, we computed the rPWR and rCST combining the median β-values of ∆HbO_2_ or ∆HHb with the median β-values of ∆oxCCO, which we extracted from the GLM. In Fig. 4, we show the haemodynamics vs metabolism plot for the Right stimulation condition as an example. On the left, we show the results using the median β-values of ∆HbO_2_ and ∆oxCCO averaged across participants, while on the right we show the median β-values of ∆HHb and ∆oxCCO averaged across participants, in the mean–variance-normalized map. Magenta circles represent bNIRS channels located in the left hemisphere (# 1–8) and black crosses channels located in the right hemisphere (# 9–16). The rPWR_HbO2_ / rPWR_HHb_ axes are shown in light blue and the rCST_HbO2_/ rCST_HHb_ axes are shown in orange. Through this visualization, the map is divided into four quadrants based on the magnitude and direction of the changes in HbO_2_/HHb and oxCCO. For instance, channels in the top right quadrant exhibit a greater increase in HbO_2_ and oxCCO, and a greater decrease in HHb, leading to a high rPWR; e.g. channel 7 has z(HbO_2_) = 1, z(HHb) = 0.57, z(oxCCO) = 0.84, corresponding to rPWR_HbO2_ = 1.3 and rCST_HbO2_ = -− 0.1, and rPWR_HHb_ = 1 and rCST_HHb_ = 0.19, suggesting that during the right hemifield stimulation, the primary visual cortex (V1) is characterized by concurrent changes in haemodynamics and metabolism (high rPWR), with nearly null mismatch (low rCST). In our case, almost all the channels are located in the top right and bottom left quadrants, suggesting that functional brain activity of the visual cortex to our task is associated to consistent changes in haemodynamics and metabolism as expected in a healthy population (i.e., greater increase in HbO_2_ and oxCCO, and greater decrease in HHb in the areas responding to the experimental task; greater decrease in HbO_2_ and oxCCO and greater increase in HHb in the unstimulated areas).

**Figure 5:**
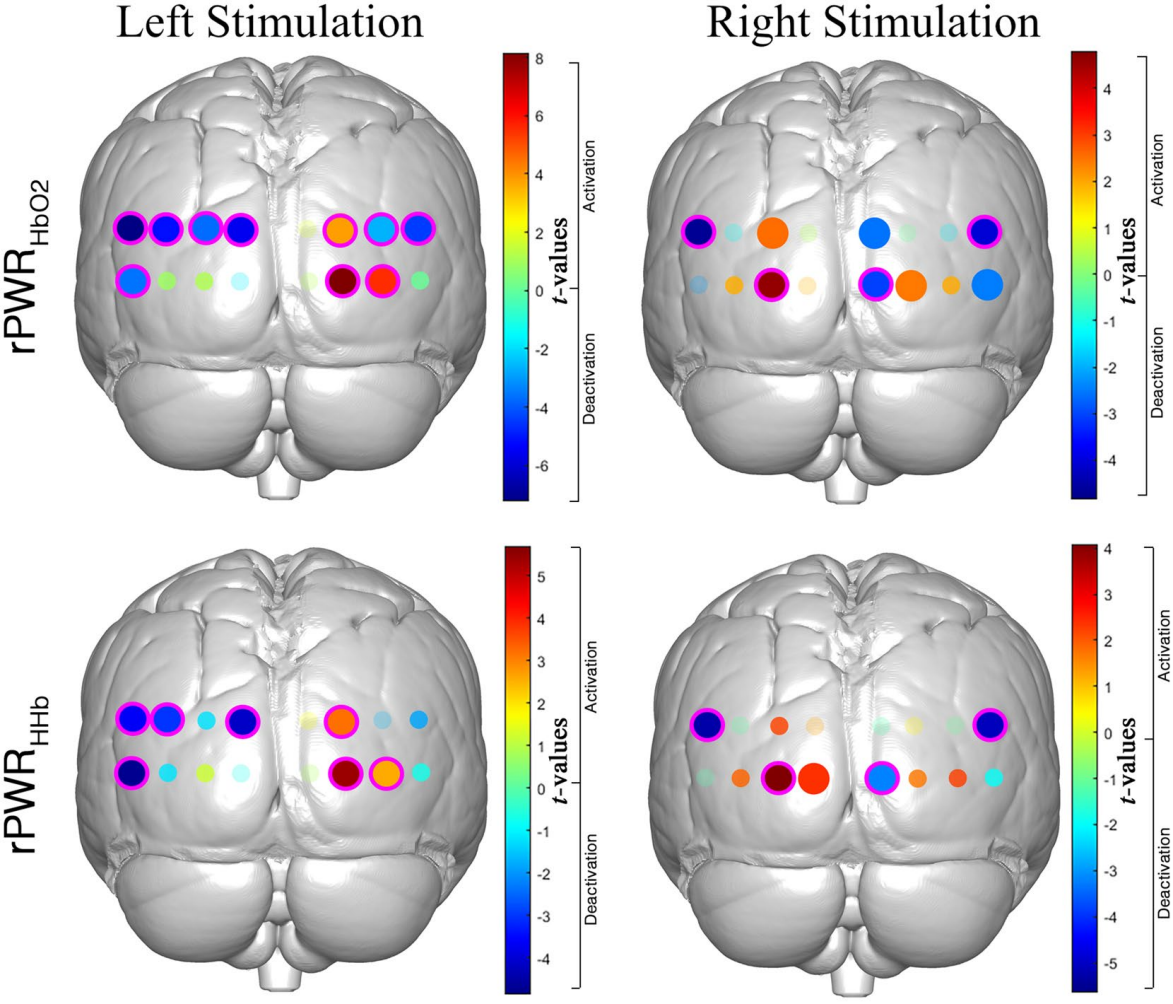
*t*-values activation maps resulting from the group-level one sample *t*-test against zero performed on the rPWR_HbO2_ (top) and rPWR_HHb_ (bottom). *t*-maps are presented for the Right (right) and Left (left) stimulation conditions. Positive *t*-values correspond to a positive association between HbO_2_/HHb and oxCCO (i.e., channels with a greater increase in HbO_2_ and oxCCO/greater decrease in HHb and increase in oxCCO) and negative *t*-values correspond to a negative association between HbO_2_/HHb and oxCCO (i.e., channels with a greater decrease in HbO_2_ and oxCCO/greater increase in HHb and decrease in oxCCO). Small shaded circles represent non-significant channels (p > 0.05), solid big circles represent significant channels (p < 0.05), and magenta circles indicate the significant channels (p < 0.05) surviving the FDR correction.

In order to localize the patterns of significant rPWR and rCST within the occipital cortex, we carried out channel-wise one sample *t*-tests against 0 on rPWR_HbO2_, rPWR_HHb_, rCST_HbO2_, rCST_HHb_. Group-level results for rPWR are shown in Fig. 5. *t*-values maps for rPWR_HbO2_ and rPWR_HHb_ of each experimental condition (Right/Left) are presented, where magenta circles indicate the statistically significant channels (p < 0.05) that survived the FDR correction.

We did not find any significant result for rCST, except for channel 10 covering the right V1-V2 regions (Supplementary Table 1) where we found a significant reduction in the coupling between HHb and oxCCO for the right hemifield stimulation, with changes in HHb exceeding the changes in oxCCO in respect to the rest of the channels. Group-level *t*-values for both rPWR and rCST are included in Supplementary Tables 3 and 4 respectively.

### Integration of bNIRS and EEG data

**Figure 6:**
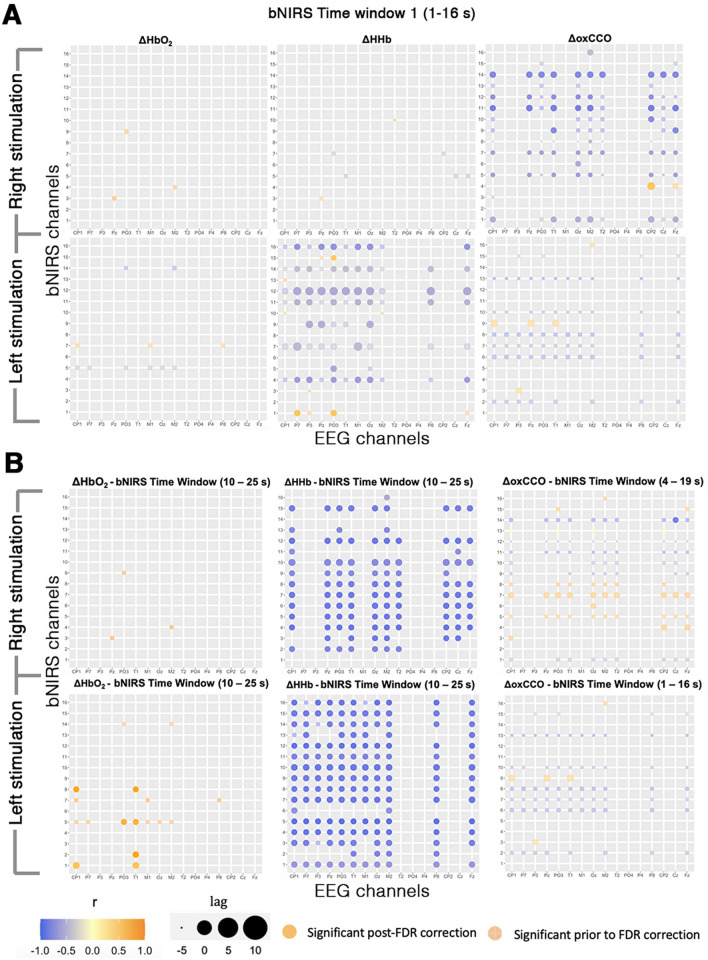
Cross-correlations between the EEG measure and the bNIRS signals at selected channels for (**A**) time window 1–16 s and (**B**) the time window where the maximum correlation between signals was observed, for the Right and Left Stimulation. For each bNIRS channel and EEG electrode combination, the significant maximum correlation (across subjects) is shown as colored circles with orange representing a strong positive correlation and purple representing a strong negative correlation, as shown by the color bar. On the y-axis, the bNIRS Channels 1–8 are channels over the left hemisphere and Channels 9–16 are channels over the right hemisphere. On the x-axis, EEG electrodes to the left of “Oz” (in the center of the x-axis) are electrodes over the left hemisphere and the electrodes to the right of “Oz” are electrodes over the right hemisphere. The transparent circles show correlations prior to FDR correction and the solid-colored circles indicate significance after FDR correction. The size of the circle represents the corresponding time lag with a positive time-lag indicating that EEG leads the bNIRS response. Note: The reader is advised that in the labelling of EEG channels, “T1” and “T2” do not refer to channels over the temporal cortex, rather the occipito-parietal region.

The cross-correlations between bNIRS and EEG were computed as described in the Methods section. For each EEG electrode and bNIRS channel combination, time window, chromophore and condition, the FDR-corrected significant correlations and their corresponding time lags were identified. In Figure g A we show the results from the earliest time window (1–16 s) during which the stimuli were being presented; Fig. 6B shows the time windows corresponding to the maximum significant correlation for each EEG-bNIRS measurement pair. The correlations are shown for each of the chromophores and conditions. The channels without any correlations are those where either the EEG gamma band activity was non-significant or the cross-correlation was non-significant after statistical analysis.

For the earliest time window (1–16 s), i.e. the time of stimulus presentation (Fig. 6A), our analysis demonstrates a predominantly negative correlation between EEG and oxCCO during the Right stimulation and a weaker negative correlation during the Left stimulation, with a median correlation of *r*_RightStim_ = -− 0.7 for the left hemisphere and of *r*_LeftStim_ = -0.65 for the right hemisphere respectively. Within this early window, correlations between EEG and haemodynamics were almost absent during the Right stimulation and weak for the Left stimulation. The EEG and the bNIRS haemodynamic signals reached the highest correlation at different time windows than the EEG and the bNIRS metabolic signals (Fig. 6B). In particular, this occurred in the final time window of 10–25 s, with the EEG and HbO_2_ showing a positive correlation but somewhat weaker for the Right stimulation, with a median correlation in the left hemisphere of *r*_LeftStim_ = 0.69. The EEG and HHb within that same window demonstrated a strong negative correlation independent to the stimuli, with a median correlation in the right hemisphere of *r*_RightStim_ = -− 0.73, *r*_LeftStim_ = − 0.83, and of *r*_RightStim_ =  − 0.84, *r*_LeftStim_ =  − 0.81 in the left hemisphere. Our results suggest that there is an earlier association between the electrical neural activity and the brain metabolic response (oxCCO) respect to the hemodynamic response (HbO_2_ and HHb) and that this happens at specific brain locations. In fact, the maximum correlation between the EEG and the oxCCO is observed at earlier time windows, that are 4–19 s for the Right stimulation (with EEG preceding the oxCCO response by 4 s) and 1–16 s for the Left stimulation (with oxCCO preceding the EEG by 2.25 s). Such negative significant correlations appear strongly and localized, only between EEG electrode Cz and oxCCO Channel 14 independently to the stimuli.

**Figure 7:**
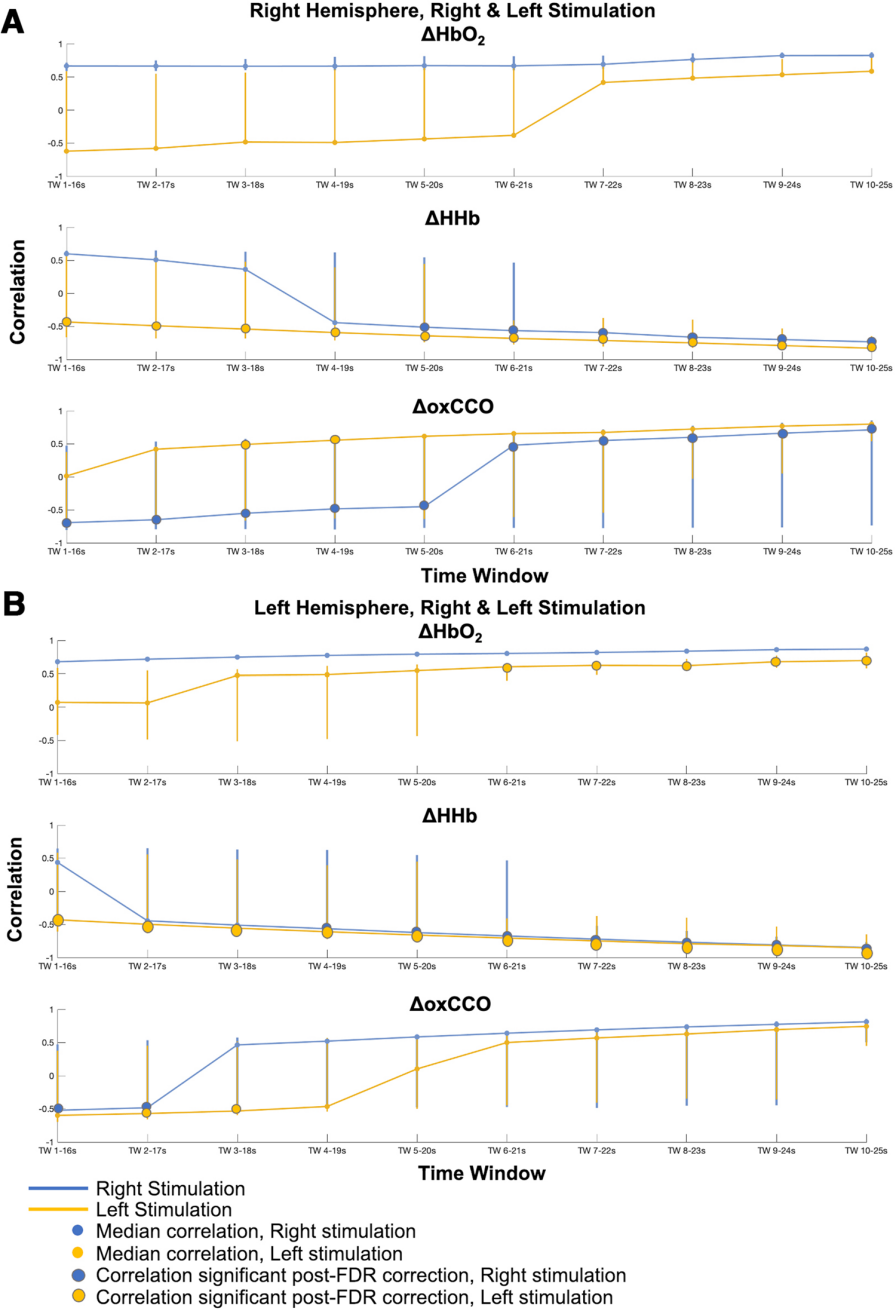
Median cross-correlations between EEG and bNIRS signals, across EEG channels, across bNIRS channels over the right and left hemispheres for all time windows from 1–16 to 10–25 s. (**A**) Median cross-correlations for Right and Left stimulations over bNIRS channels the right hemisphere, for all time windows. (**B**) Median cross-correlations for Right and Left stimulations over bNIRS channels the left hemisphere, for all time windows. The median value is shown and the lines represent minimum and maximum correlations at each time window. The correlations that were significant post-FDR correction are indicated with a larger, solid circle.

To further demonstrate how the relationship of the EEG and bNIRS correlations evolves over time, the correlations were collapsed across EEG electrodes and bNIRS channels over the left and right hemispheres to obtain a median correlation and a range (maximum to minimum) at each time window (Fig. 7).

Taken together with the results shown in Fig. 6, this further sheds a light on the relationship between bNIRS signals and neural activity. More specifically, it can be observed that: (i) HbO_2_ is seen to have a significant positive correlation in the left hemisphere during the left stimulation at later time windows (TW > 6–21 s); (ii) the EEG and HHb correlation is significant and negative and is independent to the hemisphere and stimulation. During the Left stimulation, significant correlations between the EEG and HHb can be observed as early as the first time window (1–16 s) although the maximum correlation occurs in the last time window (10–25 s); (iii) the correlations between EEG and oxCCO are significant and negative except in the right hemisphere during the Left stimulation (i.e. the contralateral hemisphere to stimulus presentation). Most interestingly however, the negative correlations are observed only in early time windows and it can be observed that a transition in the correlation between oxCCO and EEG occurs from negative to positive in the 6- 21 s time window. Supplementary Figs. 3–5 show the maximum correlations between the bNIRS signals and the EEG signal across all the windows from 1–16 s to 10–25 s.

Overall, differing time dynamics between the haemodynamic bNIRS signals and EEG and the metabolic signal and EEG can be observed with oxCCO correlating with EEG in earlier time windows and in a stimulus-dependent fashion; indicating further the different physiological information provided by oxCCO.

## Materials and methods

### Participants and experimental protocol

Thirteen healthy adults (31 ± 8 years of age, 9 males/4 females) were recruited and underwent a visual stimulation task. All participants had normal or corrected-to-normal vision and provided written informed consent in accordance with the guidelines approved by the University College London local research ethics committee. In addition, informed consent was obtained for publication of identifying images (i.e., Fig. 9C) in an online open-access publication.

**Figure 8:**
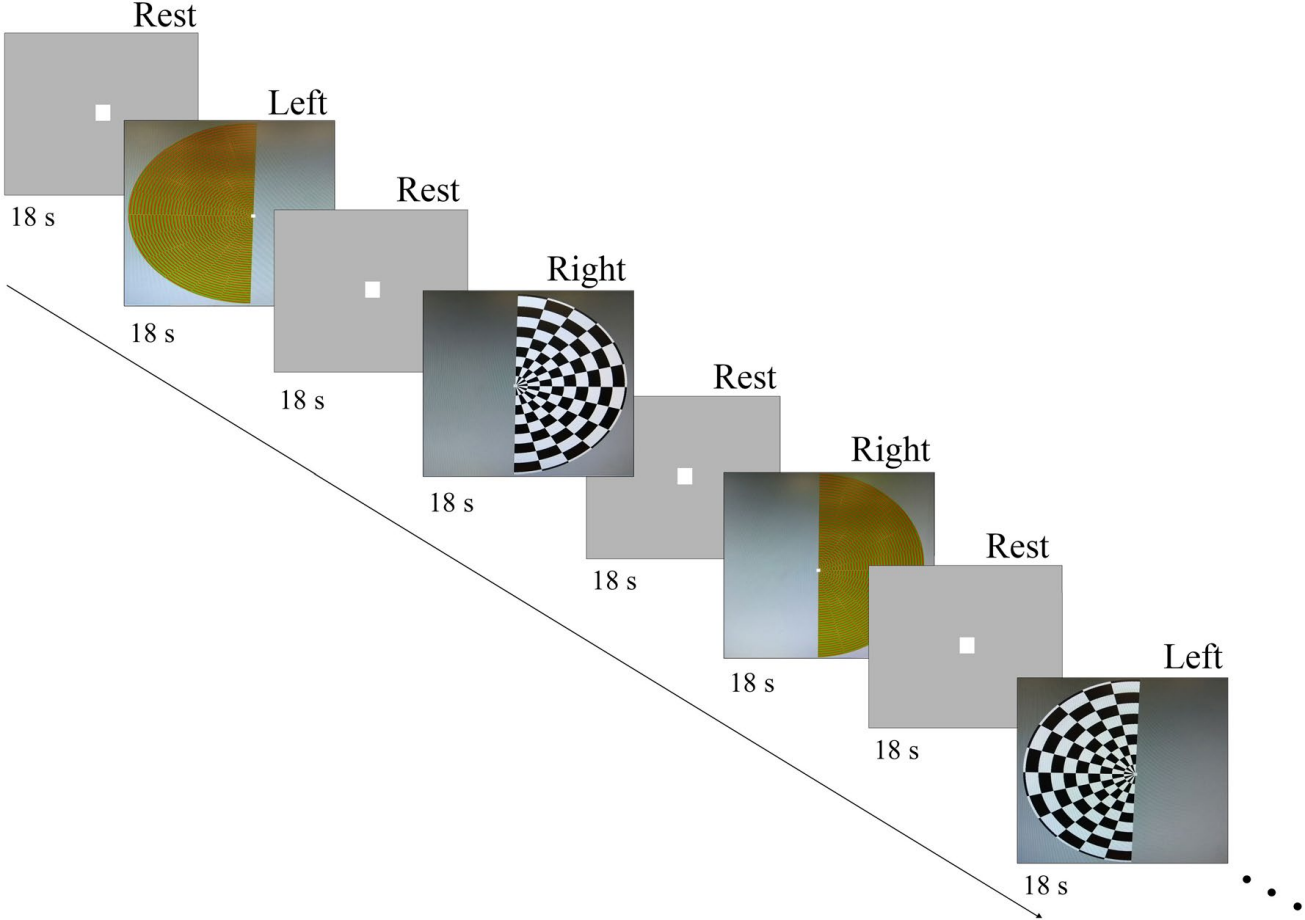
Visual stimulation task protocol. Visual stimuli comprise Right and Left hemifield reversing checkerboards spaced out by 18 s rest periods.

The visual stimulation task was designed in Psychtoolbox (MATLAB, Mathworks, USA) and consisted of right and left hemifield stimuli made of reversing black and white checkerboards (2° check size; reversal rate of 15 Hz) and isoluminant red and green checkerboards (0.25° check size; reversal rate of 2 Hz). These are known to activate the visual system pathways^19^. All participants were instructed to focus on a red fixation cross present in the centre of the screen. The visual stimulation task was structured as a block-design, with 16 blocks per condition (Right and Left) lasting 18 s spaced out by 18 s rest periods (Fig. 8). These timings correspond to a hemifield stimulation frequency of ~ 0.014 Hz. Both task and rest blocks were presented on a grey background. Right and left stimuli were alternated throughout the blocks in a fixed order (right, left, right, left, etc.) and the same sequence was used for all the participants.

### Equipment

**Figure 9:**
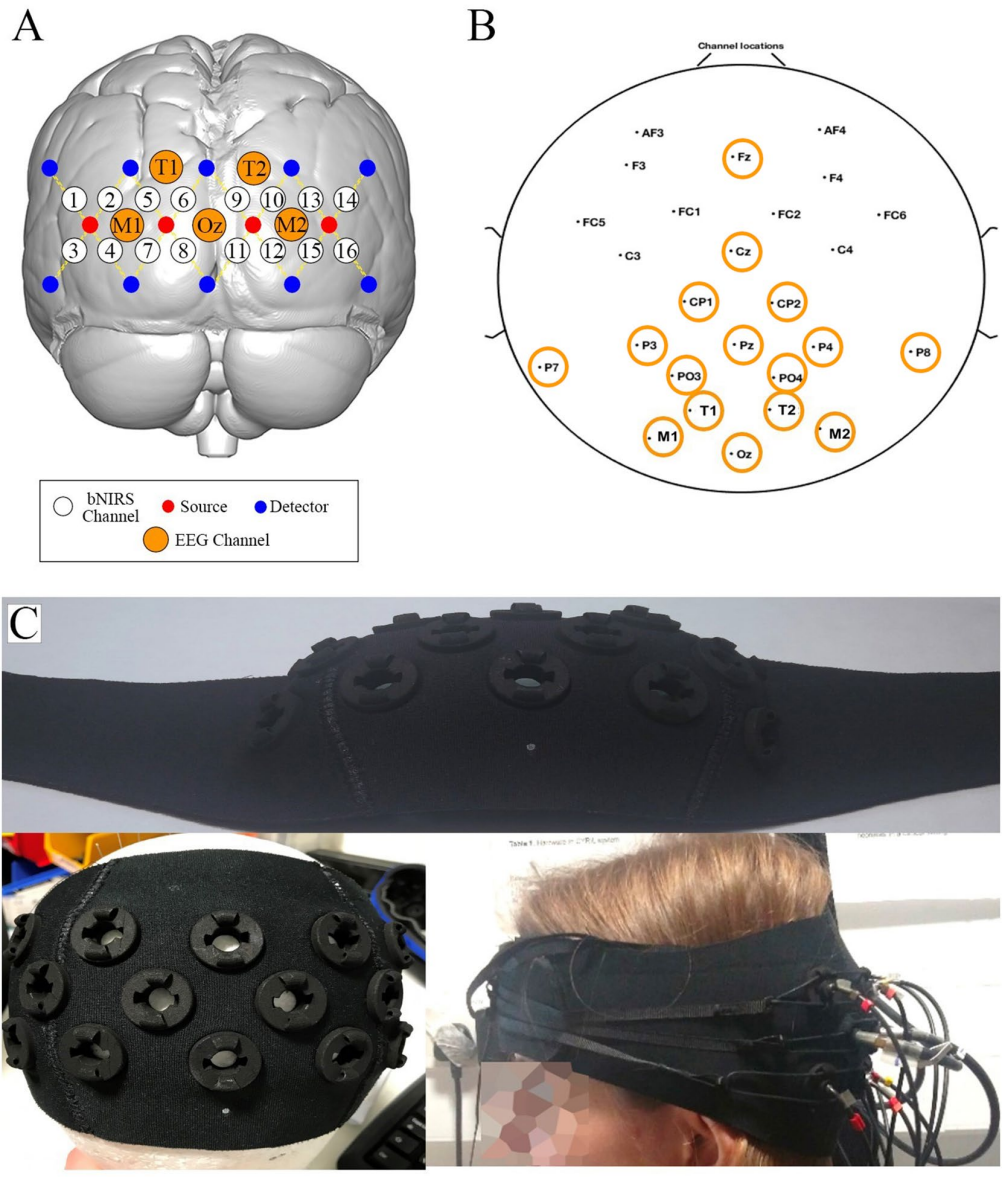
(**A**) Schematic representation of channel configuration over the visual cortex. Sources are represented by red circles, detectors by blue circles, and the 16 measurement channels by white circles. The EEG channels used for the combined bNIRS-EEG analysis are shown in orange. (**B**) Locations of the 26 EEG electrodes. The electrodes used for the combined bNIRS – EEG analysis are circled in orange. (**C**) Picture of the bNIRS probe holder. It is made of 3D printed optode holders and a neoprene band moulded to follow the curvature of the participants’ head.

Participants’ brain haemodynamics (HbO_2_ and HHb) and metabolism (oxCCO) were monitored over the visual cortex using an in-house developed multichannel bNIRS instrument (see Phan et al.^16^ for a description of the hardware). Briefly, the bNIRS system is equipped with two halogen bulbs emitting light in the NIR range (504–1068 nm) and two spectrometers (customized lens spectrographs and front illuminated CCD cameras (PIXIS512f. from Princeton Instruments)). Light is guided onto the scalp through four fiber optics bundles (sources) and the back-scattered light is collected by the spectrographs through ten fiber optics (detectors). This creates 16 measurement channels (i.e. the mid-point between a source and a detector), with a source-detector separation of 3 cm. The optodes configuration (i.e., spatial arrangement of light sources and detectors and corresponding channels) is shown in Fig. 9A. Raw intensity spectra were sampled at 0.35 Hz.

In addition to bNIRS, EEG was used to measure neural activity using a commercial wireless gel-based EEG system. The Enobio system (Neuroelectrics, Spain) was used to acquire the data and consisted of 26 measurement locations on the head which are shown in Fig. 9B. The data was sampled at a frequency of 500 Hz. The visual stimulation task in Psychtoolbox sent event markers using serial port communication to both the bNIRS and EEG systems. The bNIRS and EEG were then synchronized using these event markers.

The bNIRS fiber bundles were attached to a 3D printed optode holder (Fig. 9C) that was custom-made with a curved neoprene band to follow the curvature of the head and maximize the optical coupling. This was placed on the back of participants’ heads on top of the EEG cap to monitor the haemodynamic and metabolic changes in the occipital cortex bilaterally. Combined bNIRS and EEG headgear was custom built for simultaneous acquisition of NIRS and EEG data and consisted of a NIRS headband and an EEG cap.

In order to position the headgear in a reliable way across all participants, the measurement from the Nasion to the Inion was performed and used to identify other 10–20 anatomical landmarks along the midline such as Fz, Cz, Pz and Oz for each participant. The bNIRS-EEG headgear was then positioned such that the bottom medial detector of the NIRS headband was positioned in correspondence with the Oz landmark. Then, the EEG cap was adjusted such that the Fz, Cz and Pz electrodes were positioned over their identified anatomical locations on each subject. Following this, the locations of all bNIRS optodes and EEG electrodes were recorded through a 3D magnetic digitizer (Patriot, Polhemus, Vermont). This information was used to co-register the bNIRS channels locations onto a standard brain template. The NIRS-SPM software package^27^ was then used to obtain the MNI coordinates of the bNIRS channels and the corresponding anatomical locations. The group median MNI coordinates and the atlas-based probabilistic brain anatomical locations are reported in Supplementary Table 1. In the labelling of EEG channels, channels labelled “T1” and “T2” (Fig. 9B) do not refer to channels over the temporal cortex, rather the occipito-parietal region.

### Data analysis pipeline

In this section, we describe our suggested approach for the extraction of meaningful information from the bNIRS haemodynamic, metabolic data and EEG data. In particular, the procedure allows us to: (i) remove the most common noise components in the bNIRS signals such as physiological noise and motion artifacts to improve reliability of the extracted information; (ii) extract measures of brain haemodynamic and metabolic responses in a statistical framework overcoming the issues of modelling the brain response; (iii) combine the haemodynamic and metabolic features to evaluate the relationship between energy supply and utilization during functional brain activity and (iv) integrate the haemodynamic and metabolic signals with EEG to investigate their interrelationship and time dynamics.

**Figure 10:**
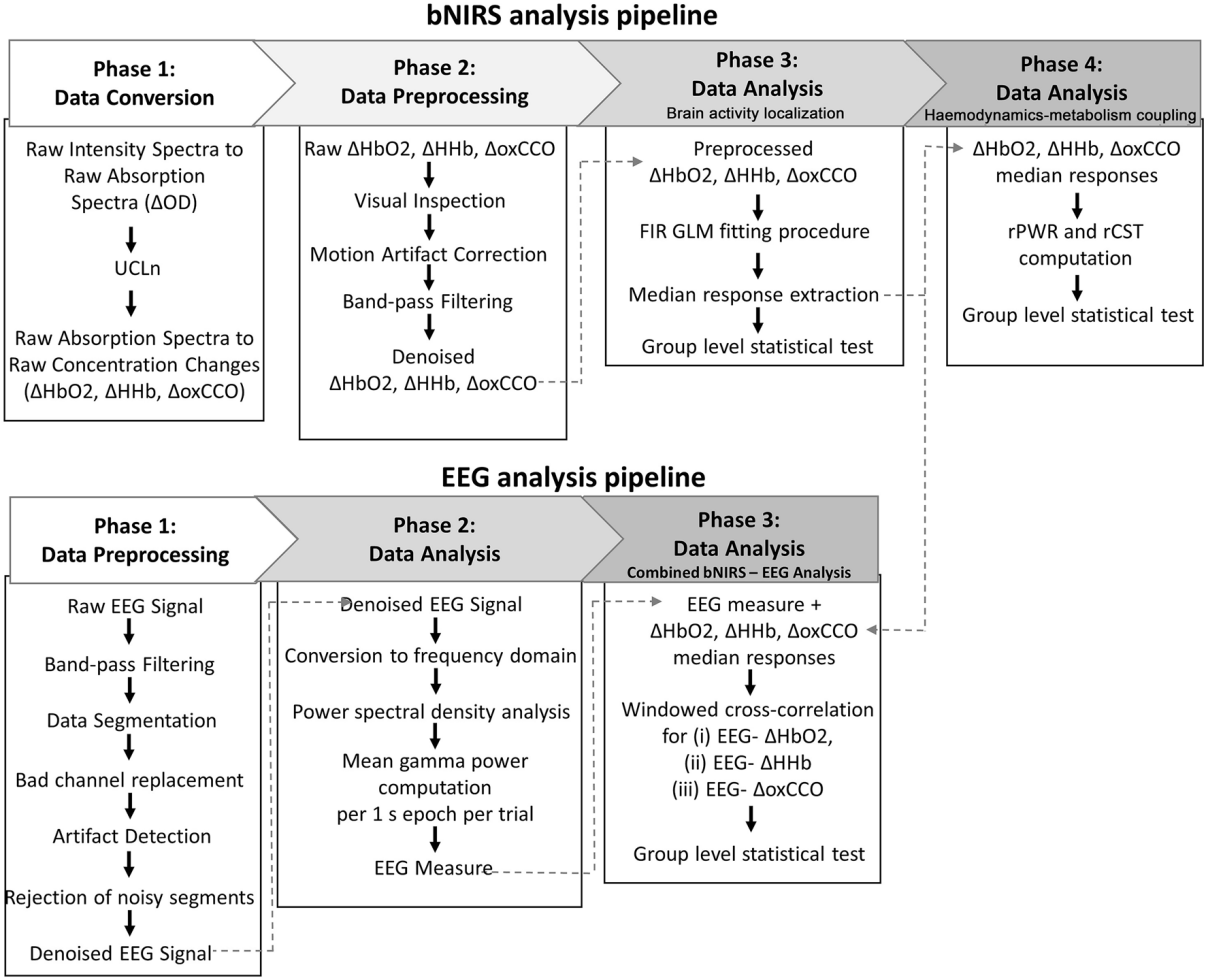
Flow chart of the data analysis pipeline for bNIRS (top) and EEG (bottom).

The data analysis pipelines for both bNIRS and EEG, as well as the procedure for the combined NIRS-EEG analysis are outlined in Fig. 10.

### bNIRS pre-processing

The bNIRS data analysis consists of the following steps.

#### Phase 1—Data conversion

Raw intensity spectra were first converted into changes in light absorbance (or optical density, ΔOD) using 120 wavelengths (from 780 to 900 nm) to minimize the cross-talk and improve the estimation of haemodynamic and metabolic signals^16^. Raw intensity spectra were first converted into changes in light absorbance (or optical density, ΔOD). The UCLn algorithm was then used to compute the changes in HbO_2_, HHb, oxCCO (ΔHbO_2_, ΔHHb, ΔoxCCO) by means of the modified Beer-Lambert Law (MBLL)^9^. Wavelength-varying differential pathlength factors (DPFs) were used, assuming DPF = 6.26 at 807 nm as described by Phan et al.^16^^16^.

#### Phase 2—Data preprocessing

Raw concentration changes in ΔHbO_2_, ΔHHb, and ΔoxCCO were first visually inspected to identify noisy channels (e.g., channels with a low signal-to-noise ration due to detector saturation or poor optical coupling) to be excluded from further analyses. This step was carried out looking at the signals both in the time-domain and in the frequency domain. In particular, the assessment of the frequency content of the bNIRS time-series through e.g. the Fast Fourier Transform (FFT) is useful not only to identify the channels, which do not include physiologically-meaningful information, but also to identify the task frequency component to preserve following the preprocessing^31^.

Artifacts due to head movements were corrected using the wavelet-based method as implemented in the Homer2 software package^32,33^, considering an interquartile range threshold (*iqr*) of 1.5. This method was chosen because it was previously demonstrated to be one of the most effective for the recovery of the haemodynamic response^34^.

In order to minimize very low and high frequency physiological noise, a band-pass filter was applied to the ΔHbO_2_, ΔHHb, and ΔoxCCO signals using a 150^th^ order FIR band-pass filter in the range [0.008 0.1] Hz. This ensured that at least 3 harmonics of the F_stim_ for the Right and Left stimulation (F_stim_ = 1/72 s =  ~ 0.014 Hz) as well as of the task presentation frequency regardless the condition (i.e., a task block every 36 s (18 s rest + 18 s task) corresponding to ~ 0.09 Hz) were included in the filter’s passband and that the filter was stable^31^. However, a low-pass cut-off frequency of 0.1 Hz does not remove the impact of Mayer waves. This is a well know issue in the fNIRS community and many different methods have been explored to reduce it. In fact, band-pass filters are not able to minimize the impact of Mayer waves as these share a common spectral range with the haemodynamic changes, and, in order to preserve the haemodynamic response, more advanced methods are needed, such as short-separation channels or monitoring blood pressure changes^35^.

### bNIRS statistical analysis

*Phase 3—Data analysis: brain activity localization*. To extract indices reflecting functional brain haemodynamic and metabolic activity and localize the brain regions responding to the experimental task, we expand the use of the GLM by using the FIR basis functions. This avoid the need of assuming any prior shape and model for the haemodynamic and metabolic responses as in the canonical GLM.

The FIR basis set is one of the most flexible basis functions which fits the measured signals with a series of contiguous boxcar functions (i.e., unitary impulses) translated over time and lasting ∆t = T/K_FIR_ each (i.e., the bin width), where T is the duration of the brain response/stimulation period and K_FIR_ is the model order^36^. This applied to each experimental condition will constitute the design matrix. A visual representation on how the design matrix regressors are generated is shown in Supplementary Fig. 1.

Here, we used a bin width ∆t = 2.88 s to match our acquisition interval (i.e. 1/0.35 Hz) with the FIR functions approximating delta-functions^36^. A β-value is then estimated using the ordinary least square estimation for each time bin and represents the weight of each basis function/delta function that best fits the input data in each time bin for each stimulation block. This results in an ‘averaged’ response T-seconds long^36^ that corresponds to the estimated haemodynamic/metabolic response to each experimental condition.

In our case, we have two experimental conditions (Right and Left) that we modelled in the design matrix. For each condition, we modelled a block lasting 36 s (i.e., our stimulation cycle) ranging from 2 s prior the stimulus onset to 34 s after the stimulus onset. Therefore, a FIR set of thirteen 2.88 s-long bins from -2 to 34 s respect to the onset was used for each task block. An example of design matrix for one participant is shown in Supplementary Fig. 1.

Hence, the resulting estimated haemodynamic/metabolic response was made of 13 β-values. The GLM-FIR procedure was applied to each channel of each participant and for ∆HbO_2_, ∆HHb, ∆oxCCO separately. We thus obtained an ‘averaged’ response for ∆HbO_2_, ∆HHb, ∆oxCCO of each channel and participant. To assess whether and where significant activations occur at the group level, we first baseline-corrected the estimated responses by subtracting the median value of the 2 s prior the stimulus onset; then, we collected the median β-value of the response in the time window 8–29 s after the stimulus onsets, which included the largest change in the responses. The median β-values computed across the 8–29 s time window for each chromophore and participant were used to run the group-level statistics and infer where statistically significant (p < 0.05) brain activity occurs. We tested for normality of our samples of median β-values using the Kolmogorov–Smirnov test at 5% significance level. All our data resulted normally distributed. We then used a one sample channel-wise *t*-test against 0 to test if significant changes in ∆HbO_2_, ∆HHb, ∆oxCCO occurred in response to the visual task, and we corrected for multiple comparisons by means of the FDR correction^37^ for number of channels.

### Integration of bNIRS haemodynamic and metabolic data

#### Phase 4 – Data analysis: metabolism-haemodynamics coupling

The analysis of the relationship between the haemodynamic and metabolic activity of the brain has the potential to unveil information regarding the energetic processes in the healthy brain in response to a particular stimulus and allow us to quantify and detect any deviations that are due to disorders and/or injury. Here, we adapt the use of the method developed by Shokri-Kojori and colleagues (2009) to compute two indices that reflect the coupling between the magnitude of brain metabolism and of activity^10^. In particular, these are: (1) the relative power (or rPWR), which reflects the extent of concurrent energy supply and utilization; (2) the relative cost (or rCST), which reflects the extent to which metabolism exceeds or falls behind the intensity of brain activity. When there is a proportional change in brain metabolism and activity, most of the common variance will be accounted for by rPWR, while the higher is the disproportion between energy supply and utilization, the more the variance will be accounted for by rCST. High values of rPWR are usually observed in metabolically demanding and high functioning brain regions (i.e., visual and default mode networks), indicating high metabolism and activity respect to the rest of the brain. High values of rCST were suggested to be related to the use of alternative metabolic pathways (e.g., aerobic glycolysis vs oxidative phosphorylation) or of alternative metabolic substrates (e.g., glucose vs ketone bodies); brain regions associated to high-level cognitive functions also showed a higher metabolic cost possibly due to use of faster but inefficient metabolic pathways^10^.

rPWR and rCST are calculated in a mean–variance-normalized (i.e. z-scored) haemodynamics-metabolism map by performing a 45° rotation of the axes (see Fig. 1d–f in Shokri-Kojori et al. (2009)). This procedure generates a rPWR axis, where the positive and negative ends correspond to high metabolism + high activity and low metabolism + low activity respectively, and a rCST axis perpendicular to rPWR, where the positive and negative ends correspond to high metabolism + low activity and low metabolism + high activity respectively. In a Cartesian coordinate system, rPWR and rCST are computed as in Eq. 1^10^:

1 $$ \left[ {\begin{array}{*{20}c} {rPWR} \\ {rCST} \\ \end{array} } \right] = \left[ {\begin{array}{*{20}c} {{\text{cos}}\left( {45^\circ } \right)} & {{\text{sin}}\left( {45^\circ } \right)} \\ { - {\text{sin}}\left( {45^\circ } \right)} & {{\text{cos}}\left( {45^\circ } \right)} \\ \end{array} } \right]\left[ {\begin{array}{*{20}c} {z\left( {activity} \right)} \\ {z\left( {metabolism} \right)} \\ \end{array} } \right] $$

The method was previously used by Shokri-Kojori et al. (2009) combining the cerebral metabolic rate of glucose (measured with FDG-PET) as the index of metabolic supply, and local spontaneous functional connectivity density as the index of brain activity (measured with fMRI). Here, we combine the median β-values of ∆HbO_2_ or ∆HHb extracted in Phase 3 as indicators of brain activity, with the median β-values of ∆oxCCO as indicators of brain metabolism. Therefore, we calculated rPWR and rCST for each channel and each participant using the z-scored median β-values across channels of ∆HbO_2_ and ∆oxCCO (rPWR_HbO2_ and rCST _HbO2_) and the z-scored median β-values of ∆HHb and ∆oxCCO across channels (rPWR_HHb_ and rCST _HHb_). For the computation of rPWR_HHb_ and rCST _HHb_, we inverted the sign of the HHb β-values so that the sign of rPWR_HHb_ and rCST _HHb_ matches with rPWR_HbO2_ and rCST _HbO2_. By plotting the median β-values of ∆HbO_2_ or ∆HHb against the median β-values of ∆oxCCO for each channel in a mean–variance-normalized map, it is possible to visualize which channel exhibits a greater association or mismatch between brain haemodynamic and metabolic activity respect to the others (e.g., Fig. 3).

In order to explore the patterns of rPWR and rCST within the visual cortex in response to the right and left hemifield stimuli, we used one sample *t*-test against 0 to localize the brain regions showing a significant positive association or mismatch between the intensity of brain activity and metabolism. Channel-wise *t*-tests vs 0 were run on the group rPWR_HbO2_ and rCST _HbO2_, and rPWR_HHb_ and rCST _HHb_, and we corrected for multiple comparisons by means of the FDR correction for number of channels.

### EEG data analysis

All EEG data were analysed in MATLAB (Mathworks, USA) using the EEGLab toolbox (Schwartz Centre for Computational Neuroscience, UC San Diego, USA) and custom-built scripts. The EEG data analysis consists of the following steps.

#### Phase 1 – Data preprocessing

The raw EEG signal was band-pass filtered between 0.1 – 100 Hz and a notch filter (48 – 52 Hz) was applied to remove artifacts due to line noise. Following this, data were segmented into 1 s epochs such that each 18 s presentation of the stimulus yielded 18 × 1 s epochs. Each 1 s segment consisted of 200 ms of the previous epoch (to serve as a baseline) and therefore baseline correction was performed after segmentation. EEG epochs were automatically rejected for movement artifacts if the signal amplitude exceeded ± 100 mV at any individual channel. Additional rejection of bad segments was performed by visual inspection of each individual epoch to remove artifacts due to eye blinks, saccadic eye movements and facial muscle movements. Channels identified as noisy during the visual inspection were interpolated i.e. replaced with an average voltage of nearby channels. All EEG data was re-referenced to the average reference.

#### Phase 2 – Data analysis

The artifact free epochs were subjected to power analysis to calculate the power spectral density (PSD) in the gamma band (25 – 45 Hz). The gamma band was chosen specifically for this analysis as previous studies have demonstrated a strong correlation between haemodynamic activity and gamma band neural activity^38–40^. The epochs were averaged across trials to obtain an averaged gamma band response per participant.

#### Phase 3 – Statistical analysis

For each participant, the maximum gamma power was obtained. T-tests were performed to compare the maximum gamma power against the baseline gamma power and FDR correction was performed to correct for multiple comparisons. The channels that had a significant gamma band activation were included in the bNIRS-EEG analysis.

The PSD results are shown in Sect. 5 in the Supplementary Material.

### Integration of bNIRS haemodynamic and EEG data

**Figure 11:**
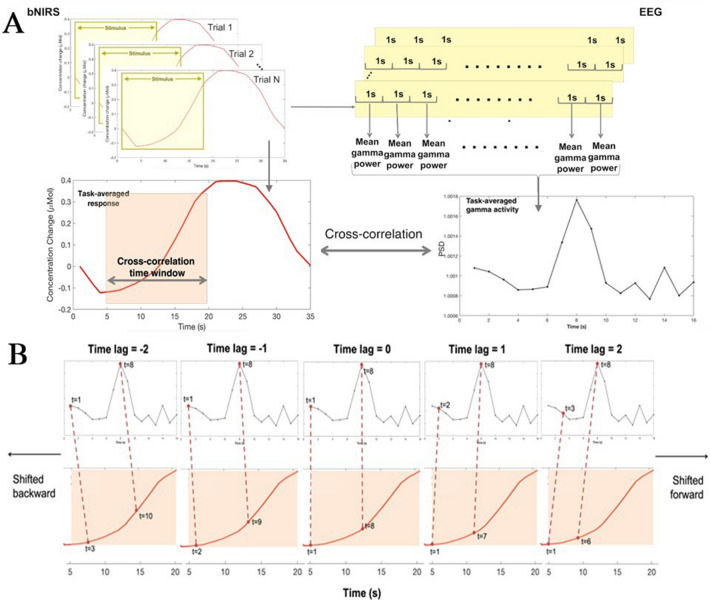
Procedure for combining the bNIRS and EEG data. (**A**) An EEG measure (mean gamma power across the task period) is derived from the EEG data that is used to cross-correlate with the bNIRS signals. The data shown here is from a single subject for the purposes of outlining the analysis procedure. (**B**) The procedure for calculating cross-correlations is demonstrated for the EEG and the NIRS data from time window 5–20 s, for selected time points, for illustrative purposes. For the positive time-lags, the cross-correlation is performed by shifting the NIRS signal forward with respect to the EEG for each time-lag. For example, for time-lag = 1, EEG time point t = 2 is correlated with bNIRS signal time point t = 1. For time-lag = 2, EEG time point t = 3 is correlated with bNIRS signal time point t = 1, i.e. the bNIRS signal is shifted forward for each positive time-lag. For the negative time-lags, the bNIRS signal is shifted back such that at time-lag = -1, EEG time point t = 1 is correlated with bNIRS time point t = 2. For time-lag = − 2, EEG time point t = 1 is correlated with bNIRS time point t = 3, i.e. the bNIRS signal is shifted backwards for each negative time-lag.

In order to investigate the relationship between the haemodynamic and metabolic signals with underlying neural activity, a combined bNIRS-EEG analysis was performed. From here on EEG channels will be referred to as electrodes and bNIRS channels will be referred to as channels. This involved first deriving an appropriate EEG measure from the EEG data to match the sampling rate of the bNIRS signals. The EEG data were thus segmented as described previously and following this, for each of the 18 × 1 s epochs that formed one stimulus presentation/one trial, the mean PSD in the gamma band of each epoch was calculated. This measure was consequently averaged across trials to obtain a measure of mean gamma power at each electrode, for each participant. This procedure is outlined in Fig. 11. The mean PSD in the gamma band was used to perform one-sample t-tests to assess whether there was a statistically significant change in gamma power during the task with respect to the baseline. Only the electrodes that were statistically significant (p < 0.05; FDR corrected for multiple comparisons) were included in this analysis.

The bNIRS data were up-sampled to 1 s and cross-correlations between the task-averaged bNIRS signals (HbO_2_, HHb and oxCCO) and the EEG measure were performed using a sliding window approach. More specifically, the EEG measure was cross correlated with 15 s of the bNIRS signals in time windows from 1 – 16 s to 10 – 25 s in 1 s increments. This yielded 10 time windows of cross-correlations.

In the current analysis, the bNIRS channels were correlated with selected EEG electrodes that were located over the occipital, parietal and central-parietal regions and nearest to the bNIRS optodes. These channels have been indicated on Fig. 9B with orange circles.

Cross-correlations provide a measure of similarity between two time series where one time series *x(t)* may be related to another time series *y(t)* by a set time-lag or time-delay. At each time-lag, to obtain the cross-correlation between the two time-series, *y(t)* is shifted along and correlated with *x(t)* at each time point to obtain a correlation at each time-lag. Correlations are calculated both for positive and negative time-lags. In order to obtain positive time-lags, *y(t)* is shifted forward with respect to *x(t)* and to obtain negative time-lags, *y(t)* is shifted backwards with respect to *x(t).* The cross-correlation for time series *x(t)* and *y(t)* (where t = 0, 1, 2,…, N) is calculated using the following formula with the time-lag equal to *d*.

$$ r\left( d \right) = \frac{{\sum \left[ {x\left( t \right)*y\left( {t - d} \right)} \right]}}{{\sqrt {\mathop \sum \nolimits_{t} \left( {x\left( t \right)} \right)^{2} } *\sqrt {\mathop \sum \nolimits_{i} \left( {y\left( {t - d} \right)} \right)^{2} } }} $$

where $\stackrel{-}{x}$ and $\stackrel{-}{y}$ are the means of *x(t)* and *y(t)* respectively and *d* = 0, 1, 2,…, N.

The cross-correlation between the two time-series yielded correlations between the pair of signals at a range of different time-lags. A positive time-lag indicated that the EEG time-series led the bNIRS signal and vice-versa for a negative time-lag. For each time window, for each subject, the maximum correlation and its corresponding time lag were identified. Bootstrapping was performed in order to reduce correlations that would occur due to the presence of autocorrelation in the bNIRS and EEG signals by randomly selecting and cross-correlating unmatched pairs for both signals (for example, the EEG signal of Subject 1 was correlated with the bNIRS signal of Subject 5). This procedure was repeated 121 times and a random sample of 11 subjects was selected to create a null distribution. Wilcoxon’s sign rank non-parametric test was used to compare the maximum correlation with the null distribution to assess the significance of the correlation. Following this, the FDR correction procedure was applied to correct for multiple comparisons^37^ for number of channels. The significant correlations after FDR correction were used to obtain the maximum cross-correlations for each time window, stimulation, chromophore (HHb, HbO_2_ and oxCCO) and channel combination. After FDR correction, for each chromophore and stimulation, the time window within which the chromophore had a maximum correlation with the EEG measure were identified.

## Discussion

In this study, we presented an analysis pipeline (Fig. 10) for the preprocessing and processing of both the bNIRS and EEG measurements. We demonstrated the use of the pipeline on data recorded at multiple locations in the visual cortex in a cohort of healthy adults, and showed how multichannel bNIRS can be used successfully to image both brain oxygenation/haemodynamics and metabolism simultaneously with EEG. The integration of oxygenation/haemodynamic and metabolic measures allows quantification and localization of brain activity and the subsequent integration with neural signals provides further insight into neurovascular coupling mechanisms. In particular, we showed that both the oxygenation/haemodynamic and metabolic signals carry information about task-related functional activity of the brain, encoding the stimulation frequency of our task as highlighted by the power spectral analysis (Fig. 1B). The preprocessing steps that we proposed resulted to be effective in minimizing the very low and high frequency noise in the bNIRS signals, while preserving the stimulation frequency (Fig. 2). In fact, when looking at the averaged time-series, the typical patterns of brain activity can be observed, with concurrent increase in HbO_2_ and oxCCO and decrease in HHb in response to the visual stimulation task (Fig. 3).

Within this framework, we expanded the use of FIR-based GLM to the analysis of both HbO_2_ and HHb, and oxCCO, thus providing new methods to extract information about both the oxygenation/haemodynamic and metabolic activity of the brain. Previous studies employing bNIRS to look at functional brain activity mainly used conventional analytical approaches to localize brain activity. For instance, Kolyva et al.^6^ and Phan et al.^16^ used the block-averaging method combined with *t*-tests to localize significant changes in HbO_2_, HHb, and oxCCO in the frontal lobe. However, the GLM was suggested to be a more powerful tool than block-averaging and has become increasingly popular for the statistical analysis of fNIRS data. Therefore, here, we moved beyond the previous basic approaches and proposed the use of GLM-based method for the analysis of bNIRS data. Thus far, flexible basis sets are recommended as, to date, there is no analogous of the HRF model for oxCCO. Model-free GLM was previously used by de Roever et al.^29^ for bNIRS, where a series of Gaussian basis functions were employed to reconstruct the haemodynamic and metabolic responses to a working memory task. However, the Gaussian function might not be optimal to model the asymmetries often seen in the haemodynamic response or for brief stimuli^41^. Hence, in this paper, we proposed a similar approach that includes the use of the most flexible basis sets for the GLM, i.e. the FIR basis functions, which allow the estimation of any arbitrary shape of the haemodynamic and metabolic responses^42^. We demonstrated that typical patterns of brain activity can be properly recovered by the FIR-based GLM and that clear and concurrent changes in ΔHbO_2_, ΔHHb, and ΔoxCCO in response to the right and left hemifield stimuli can be observed (Fig. 3). We also used the GLM to localize where significant brain haemodynamic and metabolic changes occur within the visual cortex in response to the visual task (Fig. 3). Group-level results revealed widespread significant increases in ΔHbO_2_ across all the channels, particularly for the Right condition (100% of the channels for Right, 69% for Left), while changes in ΔHHb and ΔoxCCO were somewhat more localized (ΔHHb: 81% of the channels for Right, 75% for Left; ΔoxCCO: 75% of the channels for Right, 75% for Left), in agreement with previous studies^16^. Defining cortical activation as an increase in ΔHbO_2_, decrease in ΔHHb and increase in ΔoxCCO, statistically significant activation (p < 0.005, FDR corrected) occurred in the V1, V2, V3 regions of the occipital cortex (BA 17, 18, 19 respectively; see Supplementary Tables 1 and 2) as expected for a visual stimulation task (Fig. 2). However, while activation in the contralateral hemisphere is expected^43^, we observed significant changes both in the contralateral and ipsilateral hemisphere. Ipsilateral activations might be explained by previous findings of cortical visual areas containing the representation of the ipsilateral visual hemifield as well^44^. In addition, the Right stimulation elicited more widespread changes, particularly in ΔHbO_2_ (Fig. 3, top), respect to the left stimulation (Fig. 3, bottom). This might possibly be related to ΔHbO_2_ being more prone to systemic interferences^7^ and/or to participants not looking consistently at the centre of the screen or assuming an off-centred position. However, not having external measures of visual attention and gaze (i.e., eye-tracking), in this study we could only reply on participants’ compliance to follow the instructions. In future studies, we will further expand our multimodal setup by including additional monitors of systemic physiology and participants’ behavior such as eye-tracking or ElectrOoculography/EEG to track saccadic eye movements and to improve data interpretation.

In order to leverage the advantage of bNIRS of providing simultaneous measures of brain activity and metabolism, we expanded and tested the use of two indices (rPWR and rCST) to integrate our haemodynamic and metabolic measures. These were previously developed by Shokri-Kojori and colleagues (2019) and used on FD-PET and fMRI data. More precisely, rPWR expresses the strength of the proportional change between brain activity and metabolism, while rCST quantifies the extent of the mismatch between brain activity and metabolism (i.e. how much metabolism exceeds activity and vice versa). In the study by Shokri-Kojori et al. (2009), rPWR and rCST were used to classify brain regions and resting-state networks based on the differences in the coupling metabolism-activity. For example, visual and default mode networks resulted in having the highest rPWR, being the most metabolically demanding regions, while the fronto-parietal networks showed the highest rCST, probably due to the higher metabolic cost of regions supporting high-level cognitive functions. Here, we demonstrated the feasibility to use rPWR and rCST during functional brain activity of the visual cortex by integrating measures of brain oxygenation/haemodynamics and metabolism extracted from HbO_2_, HHb and oxCCO. In case of this particular passive visual stimulation and healthy cohort, we expected to observe concurrent and greater increase in HbO_2_ and oxCCO and greater decrease in HHb in the regions involved in the task, with a balanced proportionality of the magnitude of the changes between these measurements. The rPWR and rCST have the potential to give insights into these proportional or disproportional changes between chromophore and thus into normal or different brain functioning.

Our results (Fig. 5) showed that the V1, V2, and V3 regions in the contralateral hemisphere to the stimulation exhibited the highest rPWR, meaning that those regions had the most coherent and highest changes in brain haemodynamic and metabolic activity respect to the rest of the channels. In particular, rPWR_HbO2_ and rPWR_HHb_ were both significantly positive in the contralateral V1, V2, and V3 regions and negative in the ipsilateral V1, V2, and V3 regions. Looking also at the group-averaged curves in Fig. 3, these results suggest that positive values of rPWR correspond to those channels with the largest and concurrent changes in HbO_2_/HHb and oxCCO in respect to the rest of the channels, while negative rPWR to channels with smaller changes. Therefore, it seems that rPWR shows higher spatial sensitivity than the GLM-based group analysis, identifying fewer and highly localized significant channels only within the contralateral hemisphere to the stimulation. These results are consistent with previous evidence that in a healthy brain high functional activity is associated with high metabolic supply, and that stronger activations to visual hemifield stimulation are typically observed in the contralateral hemisphere. The results for rCST further confirmed our hypothesis as we only found a significant mismatch between HHb and oxCCO in right V1-V2 (channel 10), with changes in HHb exceeding the changes in oxCCO when compared to the rest of the channels during the Right visual stimulation. This might reflect the “blood stealing” phenomenon or a suppression of neuronal activity as evidenced by previous fMRI studies, according to which a reduction in the BOLD response (corresponding to deoxyhaemoglobin) can be observed in the unstimulated part of the visual cortex (i.e. the ipsilateral hemisphere to the hemifield stimulus)^45^. Although in Fig. 3 it appears that the HHb decreases and oxCCO increases as in normal neuronal activation, such changes in channel 10 are smaller than in the rest of the channels, with HHb changes exceeding oxCCO. This leads to a negative rCST, which may reflect a reduction of neuronal activity in that area respect to the rest of the visual cortex (smaller increase in oxCCO/reduced metabolic supply) and the “blood stealing” process with increase in blood flow in the stimulated hemisphere (i.e. the left one for the Right condition) and reduction in blood flow/BOLD response in the unstimulated areas (smaller decrease in HHb in right V1-V2). In fact, channel 10 does not exhibit significant rPWR either (Fig. 3) while the corresponding channels covering V1 and V2 in the left hemisphere shows patterns of neuronal activation (i.e., proportional changes in HbO_2_/HHb and oxCCO). No significant differences in rCST were found in the rest of the channels, supporting our hypothesis that in a healthy brain there typically is an agreement between energy supply and utilization. We expect rCST to be a more informative parameter in case of brain dysfunctions and damage, as it can help in identifying those regions showing an abnormal relationship between brain metabolism and activity and altered energy use in disease^10^.

Moreover, in order to investigate the relationship between oxygenation/haemodynamics and metabolic activity with neural activity, we carried out a combined bNIRS – EEG analysis which computed the cross-correlation of the time-series (Figs. 6, 7; Supplementary Fig. 3–5). This analysis revealed a positive relationship between HbO_2_, an inverse relationship with HHb and a time-dynamic relationship with oxCCO. Correlations observed in early time windows for oxCCO reveal a negative relationship with oxCCO and EEG which transition to a positive relationship in the later time windows. Moreover, a stimulus-dependent relationship was also observed for oxCCO and EEG, i.e. a positive relationship in the contralateral hemisphere and an inverse relationship in the ipsilateral hemisphere. Previous work by Logothetis et al.^46^^46^ has suggested a direct relationship between neural activity and blood oxygenation. In particularly, previous studies provide evidence of a strong correlation between gamma band activity and the BOLD response^47–50^. Many other studies provide evidence of an inverse relationship between the task-related fMRI BOLD response and EEG oscillatory activity^51,52^. While the bNIRS analysis showed differences in the spatial localization of brain activity in response to right and left stimulation, the combined bNIRS – EEG analysis showed a similar difference for both conditions for oxCCO only. Furthermore, an interesting observation was the difference in the significant maximal time window between HbO_2_, HHb and oxCCO, with oxCCO displaying maximum correlations in an earlier time window to both HbO_2_ and HHb. In majority of channels and stimulus combinations for oxCCO, HbO_2_ and HHb, a positive (adjusted) time-lag was observed implying that neural activity preceded the haemodynamic and metabolic activity. However, for the Left stimulation condition, in the contralateral hemisphere post-FDR correction, a significant negative time-lag was observed implying that the metabolic activity preceded the neural activity. Taken together with time-dynamic and stimulus-dependent relationship observed for oxCCO, these results imply that metabolic activity ties with neural activity more closely, occurring earlier than the observed changes in haemodynamic activity.

Taken together, our results suggest that the fusion of the bNIRS-derived haemodynamic and metabolic measures has the potential to unveil additional and complementary information regarding the functioning of the brain that are not so obvious when looking at haemodynamic responses alone. In fact, the stimulus-dependent contralateral-ipsilateral representation is clearer when considering the integration of oxCCO with EEG measures (Fig. 6) and oxCCO with HbO_2_ or HHb (Fig. 5) rather than when looking at each measurement separately (Fig. 3). Further, our analysis integrating bNIRS with EEG provides insights into neural mechanisms. Nonetheless, our study presents some limitations. The bNIRS instrument used here has a lower sampling frequency (~ 0.35 Hz) compared to commercially available fNIRS devices and a better reconstruction of the temporal profile of the haemodynamic and metabolic responses could be achieved with higher acquisition rates. A low acquisition rate can also introduce artifacts in the low frequency band of the bNIRS signals as high frequency physiological noise, such as heart rate (~ 1 Hz) and respiration rate (~ 0.2 Hz), are undersampled. In the present study, this undersampling issue should not represent a problem as: (i) the artifacts are introduced in the whole bNIRS time courses, i.e. both in the rest and task periods. Hence, when comparing the changes during the task versus the changes during the rest, they are subtracted and their impact is reduced; (ii) the GLM analysis proposed here is based on a fitting procedure and is not amplitude-based. Hence, the artifacts should not be picked up and the inference is carried out on the slower haemodynamic/metabolic components. In this regard, further work is being done to develop a novel multichannel bNIRS system that will allow a sampling rate > 1 Hz. Additionally, the FIR-based GLM used here requires that the trials of the same condition have the same duration; also, while on the one hand the FIR basis functions provides the highest degree of flexibility and are particularly useful when the focus is to characterize the shape of the response, on the other hand flexibility comes with a cost, with a high number of parameters to estimate (one per each time point of the stimulus) which increases the risk of overfitting and decreases the degrees of freedom^53^. We plan to investigate the use of other flexible basis functions to overcome these limitations, such as the impulse response model^54^. Another limitation of this study is that we did not employ methods to account for systemic changes, particularly from a hardware perspective. In order to recover the haemodynamic and metabolic responses robustly, it is recommended to measure physiological data (e.g. heart rate, respiration, blood pressure, etc.) alongside bNIRS recordings or to use short-separation channels that sample data from the extra-cerebral compartment of the head, to regress these out and minimize their impact on the NIRS-derived brain signals^7^. This is especially important for the haemodynamic signals (oxy- and deoxyhaemoglobin) that are more prone to systemic interferences than oxCCO, whereas oxCCO is more brain specific^5,8^. In this study, we used a passive visual stimulation task that does not recruit higher-level cognitive functions, and we monitored a relatively small group of healthy individuals (N = 13). Future studies will investigate how the patterns of rPWR and rCST change in other brain regions and in response to more active and demanding cognitive tasks in larger cohorts. More interestingly, we aim to test the feasibility of these indices on bNIRS data recorded i.e. on individuals with altered metabolism and neurovascular coupling mechanisms, and on clinical populations such as in case of neuropsychiatric disorders. The feasibility of this technology for clinical neuromonitoring was previously demonstrated^55,56^. For instance, bNIRS was used to monitor cerebral perfusion and oxidative metabolism in newborns with hypoxic-ischaemic encephalopathy and to prognosticate the neurodevelopmental outcome of such infants^56^. We believe that bNIRS is highly suitable for clinical cohorts as measurement can be obtained in naturalistic situations, where patients do not have to be restricted inside a noisy scanner like for fMRI or PET, and metabolism and brain activity can be monitored simultaneously with no need of multiple testing sessions.

### Ethical approval and informed consent

All research and methods were carried out in accordance with relevant guidelines and regulations. All experimental protocols were approved by the University College London local research ethics committee. All participants provided written informed consent in accordance with the guidelines approved by the University College London local research ethics committee.

## Supplementary Information

Supplementary Information 1.

## Data Availability

The datasets generated during the current study are available from the corresponding author on reasonable request.
