# Supplementary material for: An analysis framework for the integration of broadband NIRS and EEG to assess neurovascular and neurometabolic coupling
Source: Sci Rep. 2021 Feb 17;11:3977. doi: 10.1038/s41598-021-83420-9 (PMC7889942; doi:10.1038/s41598-021-83420-9)
Supplement: Supplementary file 1 — Supplementary Information 1. [file 41598_2021_83420_MOESM1_ESM.docx]

**An analysis framework for the processing and integration of brain neuronal, hemodynamic/oxygenation and metabolic activity as measured by broadband NIRS and EEG**

*P. Pinti, M.F. Siddiqui, A.D. Levy, E.J.H. Jones, Ilias Tachtsidis*

**1. Group-average channels’ locations**

**Supplementary Table 1.** Group median MNI coordinates of the fNIRS channels averaged across the 13 participants. The anatomical areas (Brodmann Areas – BA) and the corresponding atlas-based probabilities for each channel are included. Only probabilities greater than 20% are listed.

| **Ch. number** | **MNI coordinates** | | | **BA-anatomy** | **Probability** |
| --- | --- | --- | --- | --- | --- |
|  | **x** | **y** | **z** |  |  |
| 1 | -49 | -82 | 22 | 39 – Angular gyrus, part of Wernike’s area  19 – V3 | 0.33  0.67 |
| 2 | -37 | -91 | 23 | 19 – V3 | 0.91 |
| 3 | -48 | -87 | 3 | 19 – V3 | 0.84 |
| 4 | -37 | -97 | 3 | 18 – Visual association cortex (V2) | 0.89 |
| 5 | -24 | -100 | 21 | 17 – Primary visual cortex (V1)  18 – Visual association cortex (V2) | 0.40  0.57 |
| 6 | -11 | -103 | 22 | 18 – Visual association cortex (V2)  17 – Primary visual cortex (V1) | 0.28  0.72 |
| 7 | -24 | -105 | 3 | 17 – Primary visual cortex (V1) | 0.88 |
| 8 | -12 | -108 | 4 | 17 – Primary visual cortex (V1) | 1 |
| 9 | 11 | -101 | 20 | 18 – Visual association cortex (V2)  17 – Primary visual cortex (V1) | 0.44  0.56 |
| 10 | 23 | -100 | 21 | 17 – Primary visual cortex (V1)  18 – Visual association cortex (V2) | 0.44  0.56 |
| 11 | 10 | -105 | 4 | 17 – Primary visual cortex (V1) | 1 |
| 12 | 23 | -105 | 4 | 17 – Primary visual cortex (V1) | 0.96 |
| 13 | 37 | -91 | 22 | 18 – Visual association cortex (V2)  19 – V3 | 0.32  0.68 |
| 14 | 49 | -81 | 22 | 39 – Angular gyrus, part of Wernike’s area  19 – V3 | 0.38  0.62 |
| 15 | 36 | -97 | 3 | 18 – Visual association cortex (V2) | 0.84 |
| 16 | 48 | -86 | 1 | 18 – Visual association cortex (V2)  19 – V3 | 0.24  0.76 |

**2. FIR design matrix computation**

In the FIR-based GLM approach, the fNIRS data are fitted with a series of regressors made of contiguous boxcar functions (i.e., unitary impulses) translated over time and lasting ∆t=T/K_FIR_ each (i.e., the bin width), where T is the duration of the brain response/stimulation period and K_FIR_ is the model order. β-values are then estimated for each time bin (Supplementary Figure 1 A) constituting the ‘averaged’ response. In our design matrix, we modelled the two experimental conditions of interest, i.e. right and left visual hemifield stimuli, considering a task block 36 s long (Supplementary Figure 1 B).


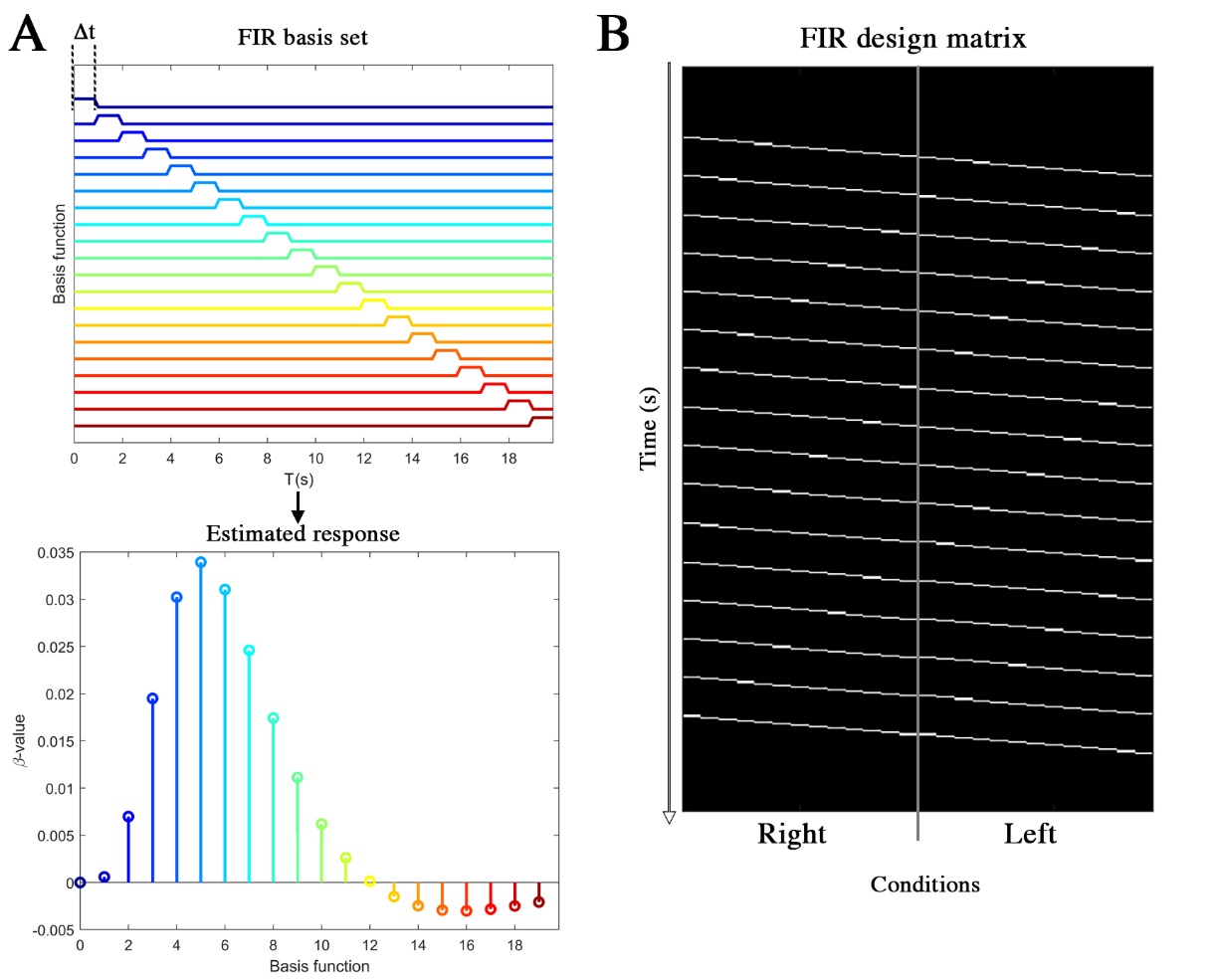


**Supplementary Figure 1.** **(A)** **FIR basis functions (top) and corresponding estimated response (bottom).** Contiguous boxcar functions translated over time with duration ∆t are used to fit the data and estimate the corresponding weight of each basis function (β-value). **(B)** **Design matrix for the visual task, composed of two experimental conditions (Right and Left hemifield).** Each stimulation (36 s) was modelled with 13 FIR functions (∆t=2.88 s).

**3. Group-level GLM results**

**Supplementary Table 2. Group-level *t*-test results for the comparisons Right > 0 and Left > 0 for each channel and chromophore.** Positive t-values indicate a significant increase in the chromophore concentration while negative-values indicate significant decreases. Statistically significant results (p<0.005, FDR corrected for multiple comparisons) are marked with asterisks.

|  | **Right > 0** | | | | | | **Left > 0** | | | | | |
| --- | --- | --- | --- | --- | --- | --- | --- | --- | --- | --- | --- | --- |
|  | ∆**HbO2** | | ∆**HHb** | | ∆**oxCCO** | | ∆**HbO2** | | ∆**HHb** | | ∆**oxCCO** | |
|  | ***t*** | **p** | ***t*** | **p** | ***t*** | **p** | ***t*** | **p** | ***t*** | **p** | ***t*** | **p** |
| **Ch 1** | 5.01* | 0.00031 | -0.50 | 0.62524 | 1.58 | 0.14016 | 1.21 | 0.25011 | -1.52 | 0.15341 | 0.64 | 0.53120 |
| **Ch 2** | 6.56* | 0.00003 | -2.35 | 0.03689 | 3.98* | 0.00182 | 2.31 | 0.03924 | -2.87 | 0.01408 | 0.89 | 0.39050 |
| **Ch 3** | 3.89* | 0.00215 | -2.48 | 0.02883 | 5.71* | 0.00010 | 2.59 | 0.02387 | -1.43 | 0.17692 | 2.61 | 0.02268 |
| **Ch 4** | 5.81* | 0.00008 | -4.00* | 0.00177 | 4.90* | 0.00036 | 4.03 | 0.00167 | -4.66* | 0.00055 | 4.91* | 0.00036 |
| **Ch 5** | 5.30* | 0.00019 | -3.70 | 0.00304 | 4.03* | 0.00166 | 1.88 | 0.08481 | -3.97* | 0.00185 | 3.48 | 0.00456 |
| **Ch 6** | 4.26* | 0.00111 | -3.57 | 0.00388 | 4.06* | 0.00157 | 1.51 | 0.15747 | -2.26 | 0.04297 | 2.53 | 0.02645 |
| **Ch 7** | 5.51* | 0.00013 | -4.97* | 0.00033 | 6.29* | 0.00004 | 3.78 | 0.00262 | -6.23* | 0.00004 | 4.89* | 0.00037 |
| **Ch 8** | 4.30* | 0.00104 | -4.74* | 0.00048 | 4.59* | 0.00062 | 2.49 | 0.02825 | -3.22 | 0.00735 | 2.63 | 0.02211 |
| **Ch 9** | 4.30* | 0.00104 | -3.31 | 0.00618 | 3.16 | 0.00824 | 3.13 | 0.00864 | -3.46 | 0.00473 | 3.53 | 0.00411 |
| **Ch 10** | 4.81* | 0.00042 | -5.11* | 0.00026 | 3.83* | 0.00238 | 3.53 | 0.00414 | -4.53* | 0.00069 | 3.91 | 0.00207 |
| **Ch 11** | 6.70* | 0.00002 | -2.83 | 0.01523 | 2.01 | 0.06777 | 3.16 | 0.00826 | -4.08* | 0.00153 | 4.16* | 0.00131 |
| **Ch 12** | 6.38* | 0.00004 | -4.44* | 0.00080 | 5.58* | 0.00012 | 5.31* | 0.00019 | -5.73* | 0.00009 | 5.86* | 0.00008 |
| **Ch 13** | 5.71* | 0.00014 | -3.72* | 0.00338 | 4.17* | 0.00156 | 2.63 | 0.02349 | -2.68 | 0.02134 | 1.48 | 0.16689 |
| **Ch 14** | 3.78* | 0.00260 | 0.66 | 0.52452 | -1.24 | 0.23880 | 1.84 | 0.09113 | -1.20 | 0.25369 | 1.62 | 0.13126 |
| **Ch 15** | 6.91* | 0.00003 | -4.45* | 0.00098 | 5.61* | 0.00016 | 4.07 | 0.00185 | -4.28* | 0.00129 | 7.66* | 0.00001 |
| **Ch 16** | 3.60* | 0.00415 | -0.88 | 0.39799 | 1.25 | 0.23704 | 3.44 | 0.00552 | -4.65* | 0.00071 | 3.39 | 0.00599 |

**4. Group-level rPWR and rCST results**

**Supplementary Table 3. Group-level *t*-test results for the comparisons rPWR_HbO2_>0 and rPWR_HHb_> 0 for each channel and condition (Right and Left).** Positive *t*-values indicate positive values of rPWR, i.e. concurrent and proportional positive changes in HbO_2_/HHb and oxCCO, while negative *t*-values indicate negative values of rPWR, i.e. concurrent and proportional reductions in HbO_2_/HHb and oxCCO. Statistically significant results (p<0.05, FDR corrected for multiple comparisons) are marked with asterisks.

|  | **Right condition** | | | | **Left condition** | | | |
| --- | --- | --- | --- | --- | --- | --- | --- | --- |
|  | **rPWR_HbO2_** | | **rPWR_HHb_** | | **rPWR_HbO2_** | | **rPWR_HHb_** | |
|  | ***t*** | **p** | ***t*** | **p** | ***t*** | **p** | ***t*** | **p** |
| **Ch 1** | -4.85* | 0.0004 | -5.65* | 0.0001 | -7.23* | 0.00001 | -3.74* | 0.0028 |
| **Ch 2** | -1.22 | 0.2464 | -1.37 | 0.1957 | -4.98* | 0.00032 | -3.09* | 0.0093 |
| **Ch 3** | -2.17 | 0.0507 | -1.47 | 0.1662 | -3.46* | 0.00474 | -4.86* | 0.0004 |
| **Ch 4** | 2.00 | 0.0684 | 1.78 | 0.1005 | 0.92 | 0.37360 | -1.14 | 0.2762 |
| **Ch 5** | 2.68 | 0.0200 | 2.07 | 0.0611 | -3.61* | 0.00356 | -1.20 | 0.2541 |
| **Ch 6** | 0.46 | 0.6538 | 1.15 | 0.2737 | -5.41* | 0.00016 | -4.25* | 0.0011 |
| **Ch 7** | 4.81* | 0.0004 | 4.09* | 0.0015 | 1.15 | 0.27129 | 1.28 | 0.2245 |
| **Ch 8** | 1.97 | 0.0721 | 2.26 | 0.0429 | -1.67 | 0.12077 | -0.70 | 0.5001 |
| **Ch 9** | -2.64 | 0.0217 | -1.46 | 0.1687 | 1.79 | 0.09880 | 1.77 | 0.1023 |
| **Ch 10** | -0.21 | 0.8357 | 0.36 | 0.7262 | 3.76* | 0.00273 | 3.40* | 0.0052 |
| **Ch 11** | -3.14* | 0.0086 | -3.50* | 0.0044 | 1.18 | 0.25973 | 0.97 | 0.3525 |
| **Ch 12** | 2.59 | 0.0238 | 1.56 | 0.1452 | 8.15* | 0.00000 | 5.70* | 0.0001 |
| **Ch 13** | -1.43 | 0.1801 | -1.23 | 0.2461 | -2.53* | 0.02789 | -1.58 | 0.1429 |
| **Ch 14** | -4.20* | 0.0012 | -5.46* | 0.0001 | -4.32* | 0.00100 | -1.87 | 0.0858 |
| **Ch 15** | 2.07 | 0.0631 | 2.12 | 0.0573 | 5.49* | 0.00019 | 2.76* | 0.0186 |
| **Ch 16** | -2.56 | 0.0263 | -2.19 | 0.0511 | 0.11 | 0.91346 | -0.68 | 0.5084 |

**Supplementary Table 4. Group-level *t*-test results for the comparisons rCST_HbO2_>0 and rCST_HHb_> 0 for each channel and condition (Right and Left).** Positive *t*-values indicate positive values of rCST, i.e. significant mismatch changes in HbO_2_/HHb and oxCCO (metabolism exceeds hemodynamics), while negative *t*-values indicate negative values of rCST, i.e. significant mismatch changes in HbO_2_/HHb and oxCCO (hemodynamics exceeds metabolism). Statistically significant results (p<0.05, FDR corrected for multiple comparisons) are marked with asterisks.

|  | **Right condition** | | | | **Left condition** | | | |
| --- | --- | --- | --- | --- | --- | --- | --- | --- |
|  | **rPWR_HbO2_** | | **rPWR_HHb_** | | **rPWR_HbO2_** | | **rPWR_HHb_** | |
|  | ***t*** | **p** | ***t*** | **p** | ***t*** | **p** | ***t*** | **p** |
| **Ch 1** | -0.46 | 0.66 | 1.58 | 0.141 | 0.82 | 0.43 | 0.76 | 0.46 |
| **Ch 2** | 0.96 | 0.36 | 1.57 | 0.143 | -0.52 | 0.62 | -1.27 | 0.23 |
| **Ch 3** | 0.80 | 0.44 | 0.24 | 0.815 | 0.25 | 0.80 | 1.97 | 0.07 |
| **Ch 4** | 0.83 | 0.42 | 0.47 | 0.646 | -1.32 | 0.21 | 0.28 | 0.79 |
| **Ch 5** | -0.33 | 0.75 | -1.80 | 0.098 | 0.97 | 0.35 | -1.68 | 0.12 |
| **Ch 6** | 1.92 | 0.08 | 0.11 | 0.911 | 1.45 | 0.17 | -0.39 | 0.70 |
| **Ch 7** | -0.94 | 0.36 | 1.75 | 0.105 | 0.37 | 0.72 | -0.49 | 0.63 |
| **Ch 8** | 2.18 | 0.05 | 1.04 | 0.320 | 0.11 | 0.91 | -1.20 | 0.25 |
| **Ch 9** | -0.01 | 0.99 | -1.75 | 0.106 | 1.03 | 0.32 | 0.39 | 0.70 |
| **Ch 10** | -1.10 | 0.29 | -4.39* | 0.001 | 0.53 | 0.61 | -0.85 | 0.41 |
| **Ch 11** | -0.44 | 0.67 | -0.99 | 0.341 | 0.17 | 0.87 | 0.47 | 0.64 |
| **Ch 12** | -0.98 | 0.35 | -0.57 | 0.582 | -1.18 | 0.26 | 0.38 | 0.71 |
| **Ch 13** | 0.63 | 0.54 | 0.49 | 0.635 | -1.01 | 0.33 | -1.56 | 0.15 |
| **Ch 14** | -1.79 | 0.10 | -0.19 | 0.851 | 0.61 | 0.56 | 0.26 | 0.80 |
| **Ch 15** | 0.82 | 0.43 | 0.47 | 0.644 | 0.55 | 0.60 | 2.59 | 0.03 |
| **Ch 16** | -0.37 | 0.72 | 0.40 | 0.694 | -1.14 | 0.28 | -0.51 | 0.62 |

**5. EEG Power Spectral Density Results.**

Supplementary Figure 2 shows the PSD measure across the stimulation period for the EEG channels of interest. The responses for the Right stimulation are shown in black while the Left stimulation are in blue. Channels that did not have a significant difference, after performing statistical tests and FDR correction, between the baseline gamma power and task-period gamma power are left blank. This PSD measure was used to perform the cross-correlations between the bNIRS and EEG signals.

**
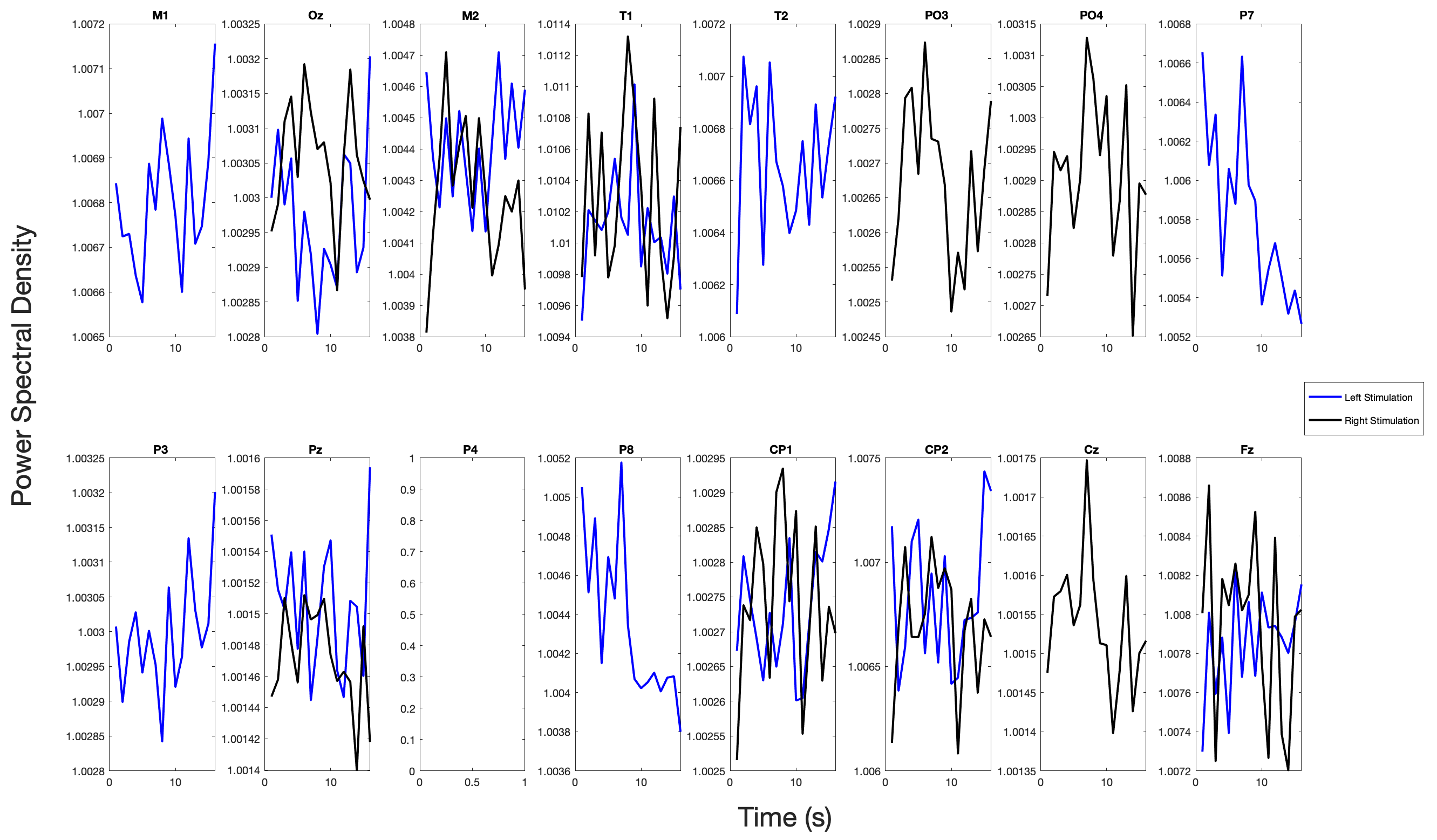
**

**Supplementary Figure 2.** **EEG PSD measure.** The PSD measure at the EEG channels of interest is shown here for both right stimulation (black) and left stimulation (blue). Those channels which did not have a significant (after FDR correction) gamma response, are left blank.

**6. Integration of bNIRS and EEG signals.**

The cross-correlation between the EEG and bNIRS signals was performed using the sliding window approach in 1 s increments. In the main manuscript, the results from the first window and the window where the maximum correlation between the signals occurred is reported. Supplementary Figures 3-5 shows the results for all three chromophores, for both stimulations, from all the time windows from 1 – 16 s to 10 – 25 s.


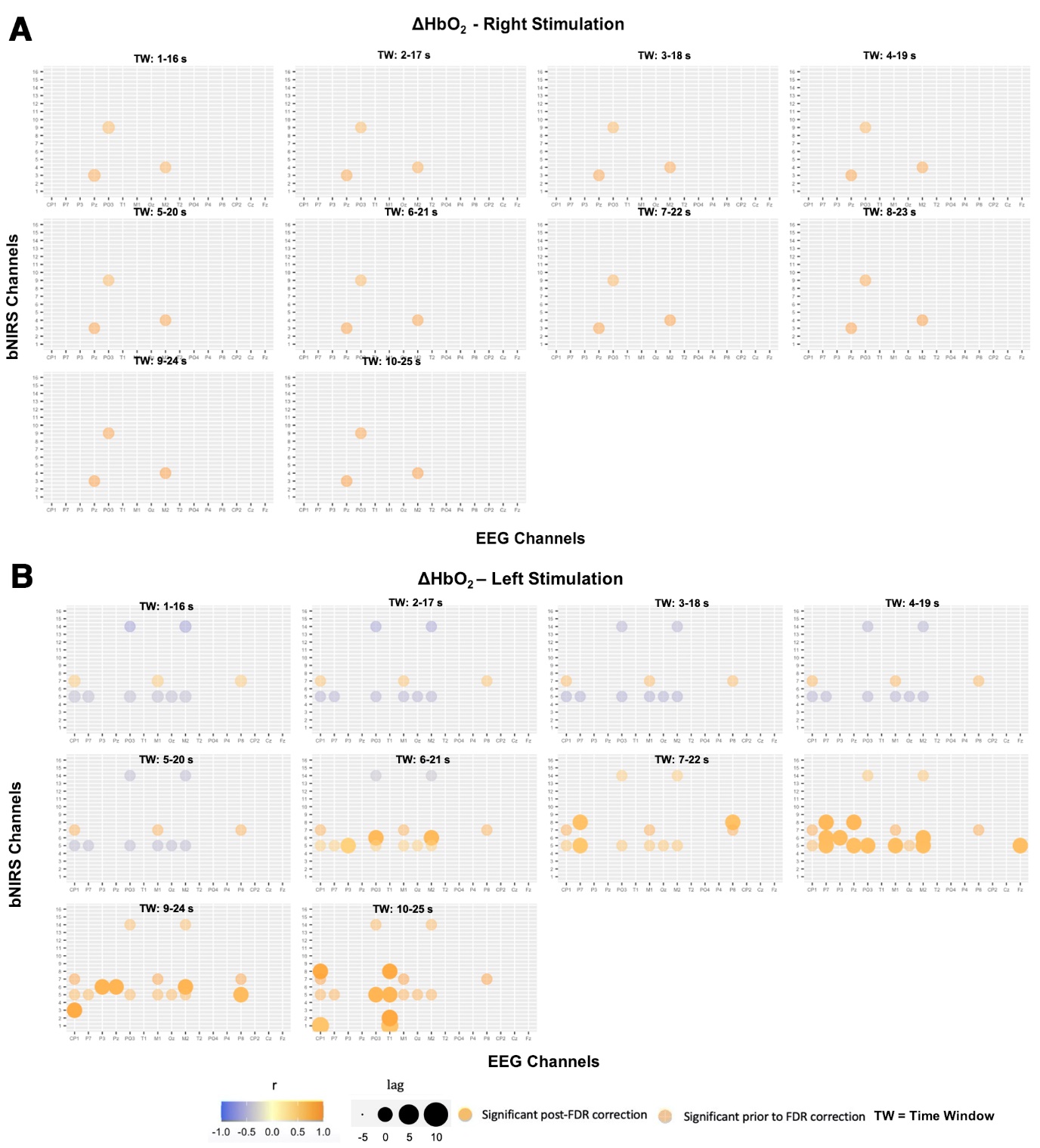


**Supplementary Figure 3.**  **Correlation between bNIRS HbO_2_ and EEG signals.** The maximum correlation between HbO_2_ and EEG signals and their corresponding lags at each of the time windows are shown for the right stimulation (A) and for the left stimulation (B).

**
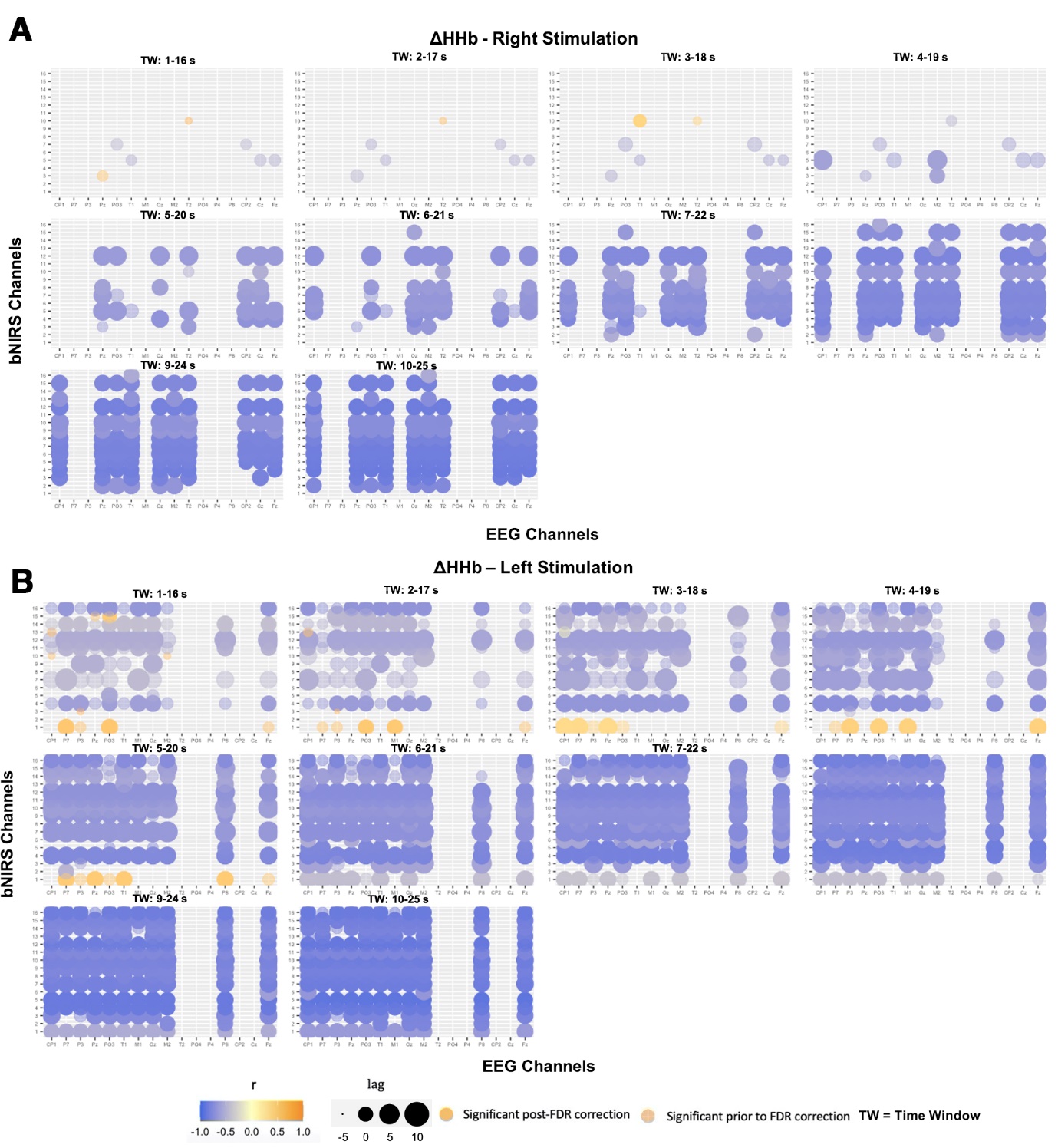
**

**Supplementary Figure 4.**  **Correlation between bNIRS HHb and EEG signals.** The maximum correlation between HHb and EEG signals and their corresponding lags at each of the time windows are shown for the right stimulation (A) and for the left stimulation (B).

**
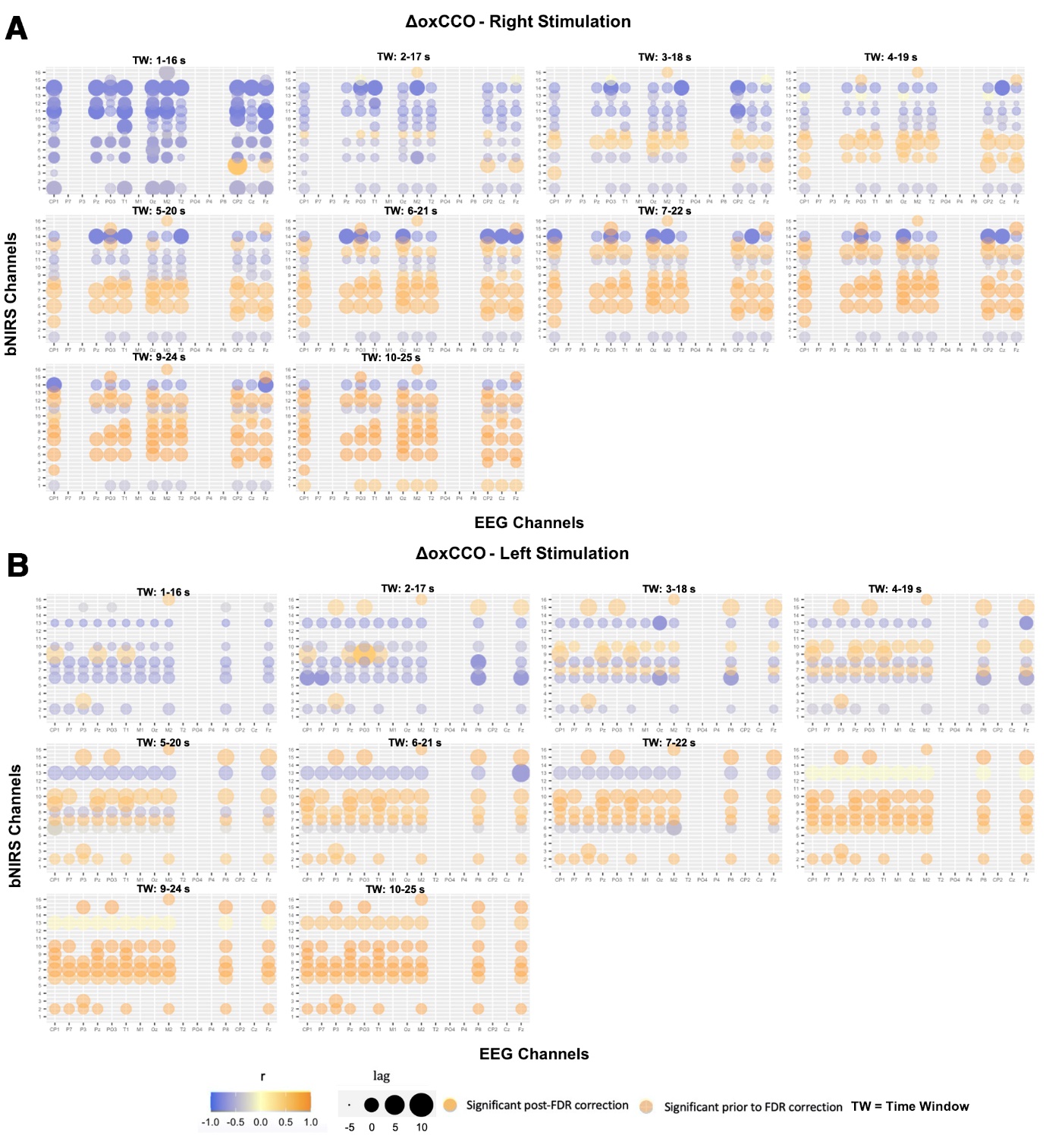
**

**Supplementary Figure 5.**  **Correlation between bNIRS oxCCO and EEG signals.** The maximum correlation between oxCCO and EEG signals and their corresponding lags at each of the time windows are shown for the right stimulation (A) and for the left stimulation (B).
